# Dasatinib overcomes glucocorticoid resistance in B-cell acute lymphoblastic leukemia

**DOI:** 10.1038/s41467-023-38456-y

**Published:** 2023-05-22

**Authors:** Jolanda Sarno, Pablo Domizi, Yuxuan Liu, Milton Merchant, Christina Bligaard Pedersen, Dorra Jedoui, Astraea Jager, Garry P. Nolan, Giuseppe Gaipa, Sean C. Bendall, Felice-Alessio Bava, Kara L. Davis

**Affiliations:** 1 Hematology, Oncology, Stem Cell Transplant, and Regenerative Medicine, Department of Pediatrics, Stanford University, Stanford, CA USA; 2 Section for Bioinformatics, Department of Health Technology, Technical University of Denmark, Kongens Lyngby, Denmark; 3 Department of Pathology, Stanford University, Stanford, CA USA; 4 M. Tettamanti Research Center, Fondazione IRCCS San Gerardo dei Tintori, Monza, (MB) Italy; 5 Baxter Laboratory, Department of Microbiology and Immunology, Stanford University, Stanford, CA USA; 6 Institut national de la santé et de la recherche médicale (INSERM), Paris, France

**Keywords:** Haematological cancer, Cancer, Cancer therapeutic resistance

## Abstract

Resistance to glucocorticoids (GC) is associated with an increased risk of relapse in B-cell progenitor acute lymphoblastic leukemia (BCP-ALL). Performing transcriptomic and single-cell proteomic studies in healthy B-cell progenitors, we herein identify coordination between the glucocorticoid receptor pathway with B-cell developmental pathways. Healthy pro-B cells most highly express the glucocorticoid receptor, and this developmental expression is conserved in primary BCP-ALL cells from patients at diagnosis and relapse. In-vitro and in vivo glucocorticoid treatment of primary BCP-ALL cells demonstrate that the interplay between B-cell development and the glucocorticoid pathways is crucial for GC resistance in leukemic cells. Gene set enrichment analysis in BCP-ALL cell lines surviving GC treatment show enrichment of B cell receptor signaling pathways. In addition, primary BCP-ALL cells surviving GC treatment in vitro and in vivo demonstrate a late pre-B cell phenotype with activation of PI3K/mTOR and CREB signaling. Dasatinib, a multi-kinase inhibitor, most effectively targets this active signaling in GC-resistant cells, and when combined with glucocorticoids, results in increased cell death in vitro and decreased leukemic burden and prolonged survival in an in vivo xenograft model. Targeting the active signaling through the addition of dasatinib may represent a therapeutic approach to overcome GC resistance in BCP-ALL.

## Abstract (web summary)

Despite playing a central role the treatment of B-cell precursor acute lymphoblastic leukaemia (BCP-ALL), resistance to glucocorticoids remains a major obstacle. Here, the authors identify activation of PI3K/mTOR and CREB pathways as a driver of GC-resistance in BCP-ALL and restore sensitivity using the multi kinase inhibitor, dasatinib.

## Introduction

Glucocorticoids (GCs) have been a mainstay in the treatment of B-cell precursor acute lymphoblastic leukemia (BCP-ALL) for decades. Poor initial response to GCs is highly prognostic of relapse^1,2^, which occurs in 15–20% of children with BCP-ALL^3^.

GCs are known to inhibit cell growth, induce cell cycle arrest, and mediate cell death through the activation of pro-apoptotic pathways. These effects are strictly dependent on the interaction of GCs with the glucocorticoid receptor (GCR). The GCR can act as a transcriptional activator or repressor through direct binding to DNA or interaction with transcription factors, such as AP-1 and NF-kB^4^. The role of GCs in human lymphocyte development has been studied primarily in the context of T-cell differentiation, where GCs antagonize negative selection^5^. Less is known about their impact on B-cell development. Studies in murine B-cells have shown that B-cells from the spleen and bone marrow express a functional GCR during B-cell development^6^.

In leukemia, GCs induce cell death even as single agents; however, not all cells are sensitive to GC-induced cell death and the mechanism behind their resistance is not well understood. We previously demonstrated that cells with activated signaling of PI3-kinase and mTOR pathways at the developmental transition between pro-B and pre-B cells are predictive of future relapse at the time of diagnosis^7,8^. Kruth et al. previously identified that suppression of B-cell developmental genes sensitizes cells to glucocorticoid treatment, hypothesizing that GCs may play a dual role in B-cell development such that they can either promote or arrest B-cells through their developmental stages^9^.

Here, we examine the glucocorticoid pathway in healthy and leukemic B-cells, and we discover a relationship between GC-resistance and B-cell development with activation of downstream PI3K/mTOR and CREB signaling. Dasatinib, a dual SRC/ABL inhibitor used clinically for the treatment of Philadelphia chromosome-positive BCP-ALL, most effectively inhibits these pathways, and its combination with dexamethasone results in increased cell death in vitro and increased survival in an in vivo model. These findings suggest dasatinib can address glucocorticoid resistance in childhood leukemia.

## Results

### Glucocorticoid receptor coordinates with B-cell developmental pathways in early human B-cells

**Fig. 1:**
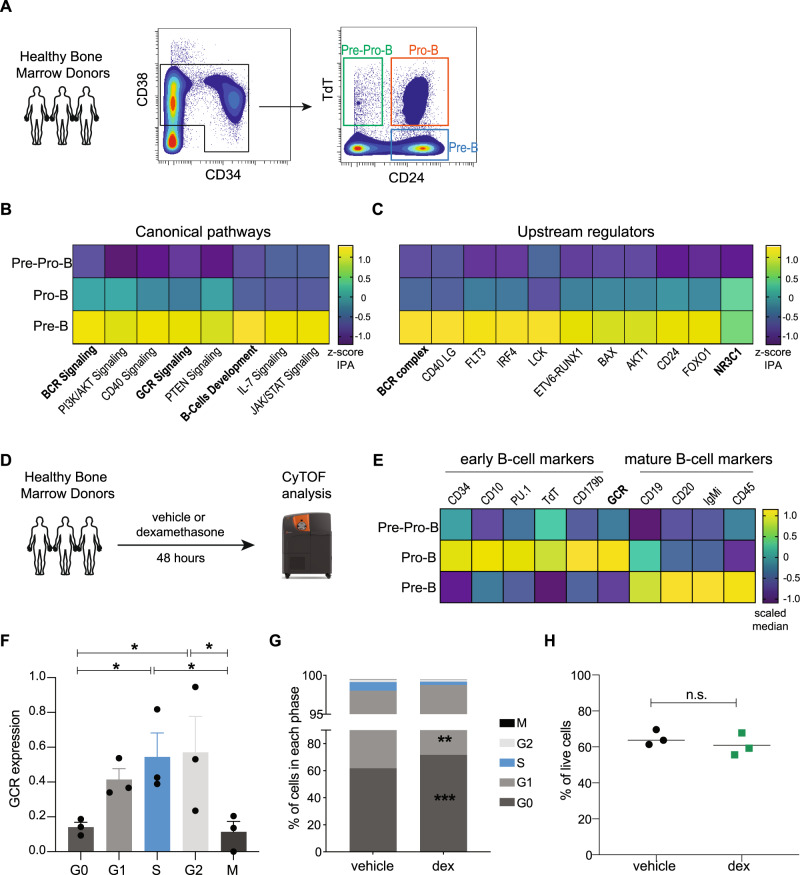
Glucocorticoid and B-cell pathways are co-expressed in healthy human B-cell progenitors. **A** Flow cytometry gating strategy used to sort B-cell developmental populations (pre-pro-B: CD34^+^/CD38^+^/TdT^+^/CD24^-^; pro-B: CD34^low^/CD38^+^/TdT^-^/CD24^+^; pre-B: CD34^-^/CD38^+^/TdT^-^/CD24^+^) in three healthy bone marrow donors for RNA-Seq analysis. **B** Canonical pathways are significantly enriched during development. Pathways highly correlated with BCR signaling were plotted; bold indicates BCR and glucocorticoid-related pathways. Heatmap is colored based on the *z*-score of IPA results. **C** Upstream regulators across development correlated with BCR complex; bold indicates BCR and glucocorticoid-related pathways. Heatmap is colored based on the *z*-score of IPA results. **D** Experimental workflow of healthy bone marrow donors analyzed by CyTOF. **E** Scaled median expression of B-cell related proteins and glucocorticoid receptor (GCR) in healthy pre-pro-B, pro-B, and pre-B cells. **F** GCR expression (Mean ± SEM) across cell cycle states (*n* = 3 healthy donors). Kruskal–Wallis nonparametric test is used to test significance (α = 0.05); G0 vs S *p* = 0.0285; G0 vs G2 *p* = 0.0358; S vs M *p* = 0.0225; G2 vs M *p* = 0.0255. **G** Mean percentage of each cell cycle phase in vehicle (ethanol) and dexamethasone (dex, 1 µM) treated cells. Paired *t*-test dexamethasone vs vehicle: G0: *p* = 0.0005, G1: *p* = 0.0021; S: *p* = 0.0789. **H** Percentage of live cells (cCASP3^-^/cPARP^-^) in vehicle (ethanol) and dexamethasone (1 μM) treated healthy cells (*n* = 3 healthy donors). Two-tailed paired *t*-test was used to test significance, *p* = 0.0742, not significant. Asterisks indicate *p* values as calculated by a two-tailed *t*-test (*p ≤ 0.5; **p ≤ 0.01; ***p ≤ 0.001; n.s. not significant). Source data are provided as a Source Data file. The experimental workflow was created with Biorender.com.

Human B-cell development is characterized by the coordination of regulatory signaling, cellular phenotype, immunoglobulin rearrangement, and cell fate^10^. To define the transcriptional dynamics dictating these developmental decisions, cells were sorted from human bone marrow (*n* = 3) to obtain pre-pro-B, pro-B, and pre-B cells, as shown in Fig. 1A and RNA sequencing was performed. Biological replicates of each sorted population clustered together and demonstrated differential gene expression across these developmental populations (Supplementary Fig. 1A). Pathway analysis revealed 399 pathways and 2672 upstream regulators differentially expressed across these populations. Expectedly, as early B-cells matured, the BCR signaling pathway was upregulated in pre-B cells compared to pro-B and pre-pro-B-cells, with the BCR complex being the most statistically significant regulator (Fig. 1B, C).

Using linear regression, we identified 88 pathways that tracked across the development with the BCR signaling pathway (*R*^2^ > 0.70; full list in Supplementary Data 1). Not surprisingly, IL-7/JAK/STAT, PI3K/PTEN, and CD40 were highly correlated with BCR signaling^11,12^ (Fig. 1B). Unexpectedly, the glucocorticoid receptor (GCR) signaling pathway was highly correlated with the BCR signaling pathway (*R*^2^ = 0.89) (Fig. 1B and Supplementary Fig. 1B), suggesting a role for the GCR pathway during B-cell development. It is notable that B cell progenitors at this stage do not express a mature B-cell receptor but instead express the pre-B-cell receptor (pre-BCR), which utilizes a similar signaling pathway and is part of the developmental selection of early B cells^13^. Indeed, within the upstream regulators, *NR3C1*, the gene encoding the GC receptor, demonstrated increased statistical significance in pro-B and pre-B-cells compared to pre-pro-B (Fig. 1C and Supplementary Fig. 1C) as well as increased gene expression (Supplementary Fig. 1D).

To determine if the GCR was also expressed at the protein level in these early B-cell populations, we analyzed three additional donors using mass cytometry (CyTOF) (Fig. 1D). GCR protein expression peaked in pro-B cells compared to pre-pro-B cells (*p* = 0.0311) and pre-B cells (*p* = 0.0243) as shown in Supplementary Fig. 1E. GCR expression followed a similar pattern of expression as other markers of early B-cell progenitors, including CD34, CD179b, TdT, CD10, and PU.1^10^ (Fig. 1E).

Given that cells at the pro-B to pre-B transition undergo periods of proliferation and cell cycle arrest^10^ and that GCR signaling regulates the cell cycle in other contexts^4,11,14^, we examined GCR expression across the cell cycle in healthy B-cell progenitors. GCR expression peaked in S-G2 phases (Fig. 1F). Exposing healthy cells to dexamethasone, we observed an increased proportion of cells in the G0 phase (*p* = 0.0005), decreased proportions in G1-phase (*p* = 0.0021), and loss of cells in S-phase (*p* = 0.0789) (Fig. 1G). As previously demonstrated, the GCR was downregulated upon dexamethasone treatment (Supplementary Fig. 1F, G)^15^. Exposing these healthy B-cell progenitors to dexamethasone did not result in increased apoptosis (Fig. 1H). These data demonstrate the upregulation of the glucocorticoid pathway as early B cells transition from pro-B to pre-B cells. Exposure to GCs induces cell cycle arrest but not apoptosis.

### Signaling pathway activation in leukemic cells surviving dexamethasone

**Fig. 2:**
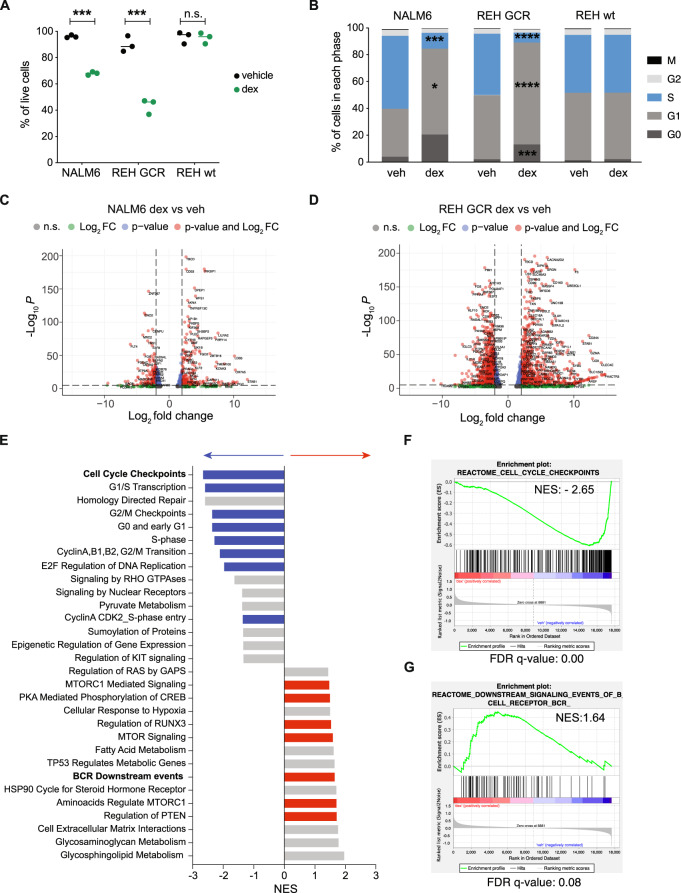
Glucocorticoid-mediated response and resistance in NALM6 and REH GCR cells. **A** Percentage of live cells (cPARP^-^/cCASP3^-^ gated cells) in vehicle (ethanol, veh) and dexamethasone (dex, 1 µM) treated conditions in NALM6 and REH overexpressing GCR (REH GCR) and REH wild type (wt). Individual plots from three independent biological replicates are shown with the median and significance was tested using a two-tailed paired *t*-test. NALM6: *p* = 0.0012; REH GCR: *p* = 0.0075. **B** Frequency in each cell cycle phase in vehicle versus dexamethasone (1 µM) NALM6, REH GCR, and REHwt cells. Asterisks indicate the significance of Fisher’s LSD test between vehicle and dexamethasone for each phase. NALM6: G1 *p* = 0.0263; S *p* = 0.0007; REH GCR: G0 *p* = 0.0003, G1 *p* < 0.0001, S *p* < 0.0001. **C** Differentially expressed genes in NALM6 cells in dexamethasone versus vehicle. The log2FoldChange (log_2_FC), *p* values, and adjusted *p* values (*p*adj) were calculated using DESeq2 default settings (alpha = 0.05, lfcThreshold = 1, *p*AdjustMethod = “BH”) and significant genes were considered the ones with *p*adj <0.05. Volcano plots were generated using EnhancedVolcano R package^61^ and default cutoffs were used: FC| > |2 and *p* value 10e^−6^. **D** Differentially expressed genes in REH GCR cells in dexamethasone versus vehicle. The log_2_FC, *p* values and adjusted *p* values (*p*adj) were calculated using DESeq2 default settings (alpha = 0.05, lfcThreshold = 1, *p*AdjustMethod = “BH”) and significant genes were considered the ones with *p*adj <0.05. Volcano plots were generated using EnhancedVolcano R package^61^ and default cutoffs were used: FC| > |2 and *p* value 10e^−6^. **E** NES (normalized enrichment score) after GSEA analysis using the Reactome database. Blue bars indicate cell cycle-related pathways downregulated in dexamethasone-treated cells, while red bars highlight BCR downstream signaling pathways upregulated in dexamethasone-treated cells. FDR <0.25 cutoff was used. **F** Enrichment plots of cell cycle checkpoints with specific NES score and FDR value **G** Enrichment plots of downstream signaling events of BCR with specific NES score and FDR value. Source data are provided as a Source Data file.

Given the coordinated co-expression of BCR and GCR pathways in healthy early B-cells and the importance of B-cell developmental state in leukemia relapse^7^, we examined this relationship in BCP-ALL cells. GCR protein expression was measured in two BCP-ALL cell lines, REH and NALM6. REH cells had no expression of GCR protein as they harbor a nonsense mutation in the *NR3C1* gene^16^ (Supplementary Fig. 2A) and accordingly did not respond to dexamethasone with any change in cell viability or the cell cycle (Fig. 2A, B), in line with previous studies linking GCR loss to GC-resistance^17,18^. We generated REH cells overexpressing GCR (REH GCR) through retroviral transduction of the *NR3C1* gene (Supplementary Fig. 2A). Unlike healthy B-cells, treating NALM6 and REH GCR cells with dexamethasone resulted in a significant reduction of live cells (NALM6 *p* = 0.0012; REH GCR *p* = 0.0075) confirming that re-introduction of the GCR to REH cells sensitizes them to GC-induced apoptosis (Fig. 2A). Dexamethasone caused a significant reduction of cells in S-phase (NALM6 *p* = 0.0007; REH GCR *p* < 0.0001) along with an accumulation of cells in G0/G1, similar to the effects observed in healthy early B-cells (Fig. 2B).

Whole transcriptome sequencing of NALM6, wild-type REH, and REH GCR cells after 48 h of dexamethasone or vehicle revealed 914 genes differentially expressed in NALM6 cells (Fig. 2C), 3190 genes in REH GCR cells (Fig. 2D) and three genes in wild-type REH cells (all genes listed in Supplementary Data 2). Gene set enrichment analysis demonstrated that cells surviving dexamethasone treatment downregulate pathways related to cell cycle regulation, DNA replication, and post-translational modifications (Fig. 2E, blue bars, Fig. 2F), and upregulate pathways related to BCR downstream signaling (Fig. 2E, red bars, Fig. 2G), including PTEN, MTORC1, and CREB signaling, suggesting that the upregulation of these signaling pathways may play a role in GC-resistance.

### Dasatinib targets GC-resistant cells

**Fig. 3:**
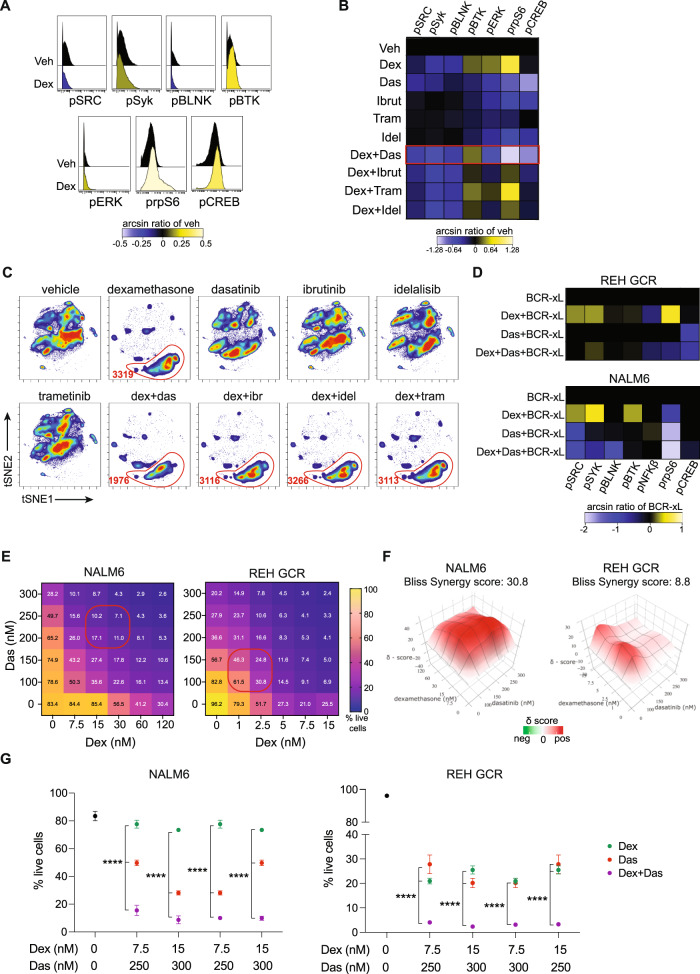
Response to combination treatment with dexamethasone and tyrosine kinase inhibitors in cell lines. **A** Expression of signaling molecules in live (cPARP-/cCASP3^-^) REH GCR cells treated with either vehicle or dexamethasone. Histograms are colored based on mean expression compared to vehicle. **B** Phosphoproteins expression in live (cPARP^−^/cCASP3^−^) REH GCR cells following treatments for 48 h. The red box indicated the most significant treatment as calculated in Supplementary 2C. **C** tSNE density plots of live REH GCR cells following treatments. In red is highlighted the population emerging after dexamethasone treatment and the numbers indicate the number of cells in the gates **D** Expression of signaling proteins in NALM6 and REH GCR live cells following treatments for 48 h. Pre-BCR signaling stimulation has been performed treating cells with IgM for 10 min and H_2_O_2_ for 5 min. The ratio of the mean expression (arcsin transformed) compared to vehicle is plotted. **E** Percentage of NALM6 and REH GCR live cells (Ann V^-^/7AAD^-^ cells) following treatment with five different concentrations of dexamethasone (dex), dasatinib (das), and combinations (*n* = 36). Red boxes highlight areas of most synergy among drug combinations. **F** Bliss synergy score (δ score) among dexamethasone and dasatinib combinations in NALM6 (left) and REH GCR (right). **G** Viability (Ann V^-^/7AAD^-^ cells) after dexamethasone (dex), dasatinib (das), dexamethasone + dasatinib (dex + das) conditions in NALM6 (left), and REH GCR cells (right). A total of *n* = 4 independent experiments are plotted as mean ± SEM. Asterisks indicate significant differences between single-drug treatments compared to the combined treatment obtained from ANOVA analysis followed by Tukey’s test (α = 0.05). NALM6: all the comparisons *p* < 0.0001; REH GCR: all the comparisons *p* < 0.0001. Veh vehicle, dex dexamethasone, das dasatinib, Ibrut Ibrutinib, Tram Trametinib, Idel Idelalisib, BCR-xL pre-BCR crosslinking. *p ≤ 0.5; **p ≤ 0.01; ***p ≤ 0.001; ****p ≤ 0.0001. Source data are provided as a Source Data file.

Transcriptomic data from cells surviving GC treatment implicated the B-cell receptor pathway in GC resistance in BCP-ALL cells. Coordination of these pathways was also suggested in the healthy transcriptomic data. REH GCR cells treated with dexamethasone demonstrated activation of prpS6, pCREB, pSyk, and pBTK (Fig. 3A), while NALM6 cells activated only prpS6 (Supplementary Fig. 2B). We tested a panel of tyrosine kinase inhibitors, in combination with dexamethasone, to inhibit this active signaling. Treating REH GCR cells with dasatinib (dual SRC/ABLi), ibrutinib (BTKi), trametinib (MEK1/MEK2i), and idelalisib (PI3Kδi) either alone or in combination with dexamethasone, we found dasatinib to be most effective to decrease the dexamethasone-induced signaling (Fig. 3B and Supplementary Fig. 2C). Evaluating surviving cells by CyTOF, we identified an emerging population after dexamethasone treatment and its combinations. Dasatinib was the only inhibitor able to reduce the number of cells in this population when combined with dexamethasone (Fig. 3C). The phenotype of dexamethasone-surviving cells is shown in Supplementary Fig. 2D, E.

Cells surviving dexamethasone are able to respond to crosslinking of the pre-BCR with activation of components of the pre-BCR signaling pathway, including pSRC, pSYK, pBLNK, pBTK, pNFKβ, prpS6, and pCREB. Adding dasatinib to dexamethasone inhibited this activation in NALM6 and REH GCR (Fig. 3D).

Based on these findings, we focused on the combination of dasatinib and dexamethasone to determine their killing effect. For both NALM6 and REH GCR cells, five concentrations for each drug for a total of 36 combinations were tested in combination. Dexamethasone concentrations differed between the cell lines as REH GCR cells are more sensitive to dexamethasone compared to NALM6 cells. The combination of dexamethasone and dasatinib resulted in more apoptosis compared to treatment with dexamethasone or dasatinib alone in a dose-dependent manner (Fig. 3E). Bliss synergy analysis revealed the combination of dasatinib with dexamethasone to be highly synergistic in NALM6 cells (mean synergy score: 30.8) and additive in REH GCR (mean synergy score: 8.8) (Fig. 3F). The dexamethasone and dasatinib combination resulted in more apoptosis in both cell lines compared to single-drug treatment (Fig. 3G). Together, these results suggest that dexamethasone resistance in BCP-ALL may be related to active signaling in the PI3K/mTOR and MEK/ERK pathways and dasatinib may best target this signaling resulting in increased apoptosis of dexamethasone-resistant cells.

### Primary BCP-ALL cells maintain developmental expression of the GCR

**Fig. 4:**
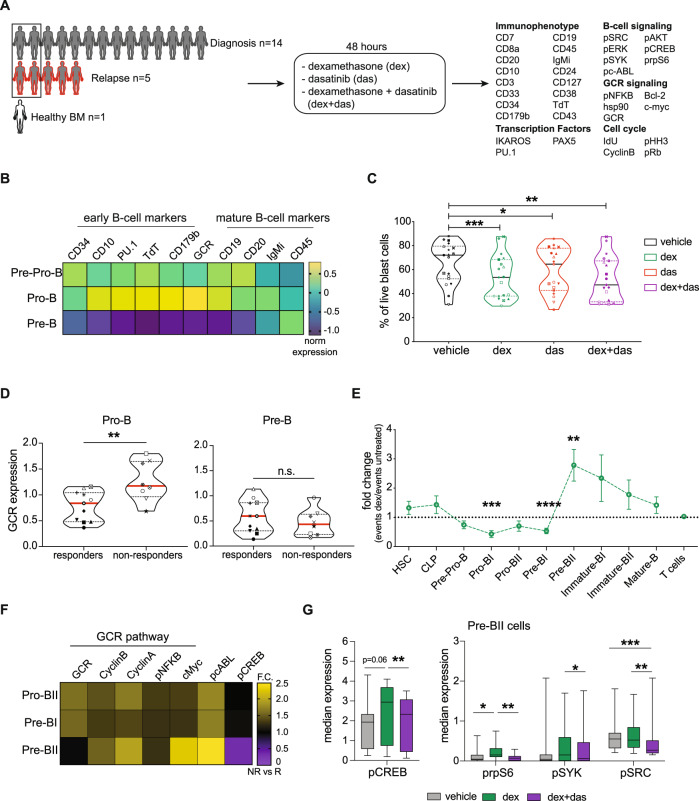
Dexamethasone-resistant cells have a distinct phenotype and signaling profile. **A** Graphical overview of the experimental approach for CyTOF analysis. **B** Normalized expression of B-cell developmental proteins and the GCR. **C** Percentage of live cells (cPARP^-^/cCASP3^-^) in vehicle, dexamethasone (dex), dasatinib (das), and combination (dex + das). Each point represents a patient sample, dotted lines indicate 25 and 75% quartiles and bold lines indicate medians for a total of *n* = 19 primary samples per condition. Asterisks indicate significant differences based on ANOVA analysis followed by Tukey’s test (α = 0.05). Dex vs vehicle: *p* = 0.0007; das vs vehicle: *p* = 0.0181; dex + das vs vehicle: *p* = 0.0011. **D** GCR expression in live pro-B and pre-B cells (cPARP^-^/cCASP3^-^) from responders (*n* = 11 primary samples with dexamethasone-induced cell death >10%) versus non-responders (*n* = 8 primary samples with dexamethasone-induced cell death <10%). Each point indicates a patient sample and is matched to panel **C**. Asterisks indicate significance based on a two-tailed unpaired *t*-test. Pro-B: responders vs non-responders *p* = 0.0065; Pre-B: responders vs non-responders *p* = 0.4030. **E** Mean fold change ± SEM of events assigned to each population in dexamethasone-treated cells compared to vehicle (*n* = 19 primary samples). ANOVA analysis followed by Fisher’s LSD test (α = 0.05) was used to identify populations that were statistically significant compared to the T-cell population. Pro-BI: *p* = 0.0001; Pre-BI: *p* < 0.0001; Pre-BII: *p* = 0.0047. **F** Fold change in protein expression between non-responders (*n* = 8 primary samples) versus responders (*n* = 11 primary samples) in a vehicle-treated condition of GCR targets. **G** Protein expression in vehicle, dexamethasone, and the combination in pre-BII classified cells. Box bars indicate mean expression in the *n* = 18 primary samples, error bars the 5th and 95th percentile and asterisks indicate significant differences based on a two-tailed paired *t*-test followed by Bonferroni correction. pCREB: dex vs veh *p* = 0.0679; dex vs dex + das *p* = 0.0098. prpS6: dex vs veh *p* = 0.0465; dex vs dex + das *p* = 0.0011. pSyk: dex vs veh *p* = 0.0756; dex vs dex + das *p* = 0.0558. pSRC: dex vs veh *p* = 0.7229; dex vs dex + das *p* = 0.0096; dex + das vs veh *p* = 0.0008. Pt10 was excluded from the analysis because no cells were classified in this population following treatment. *p ≤ 0.5 **p ≤ 0.01; ***p ≤ 0.001, ****p ≤ 0.0001. Source data are provided as a Source Data file. The experimental workflow was created with Biorender.com.

Next, we evaluated dexamethasone response and resistance in primary BCP-ALL cells from diagnosis or relapse. Nineteen primary samples underwent ex-vivo treatment with vehicle, dexamethasone, dasatinib, or the combination for 48 h (Fig. 4A). In line with the healthy bone marrow, GCR expression in leukemic cells peaked in pro-B cells and was coordinated with the expression of TdT, CD179b, CD10, and PU.1 (Fig. 4B). GCR expression was highly heterogeneous across the samples with the highest levels observed in relapse samples (Pt06R, Pt11R, Pt13R, and Pt14R) and diagnostic samples from patients with poor prognostic features: Pt16, Pt17 (*KMT2A*r), and Pt02 (Ph+ and protocol-defined poor prednisone responder) (Supplementary Fig. 3A and Supplementary Table 1).

Across the cohort, dexamethasone caused significant apoptosis (*p* = 0.0007) (Fig. 4C). However, this was not true on a per-patient basis. Nearly half of the samples (8/19) demonstrated less than 10% apoptotic cells after 48 h of dexamethasone exposure (Supplementary Fig. 3B). We defined these samples as non-responders. Interestingly, GCR expression was higher in non-responders compared to responders but only in pro-B cells (*p* = 0.0065) and not in pre-B cells (Fig. 4D), suggesting that GCR expression alone does not strictly predict response or resistance.

Ex-vivo treatment of primary cells with dasatinib alone and in combination with dexamethasone also decreased cell viability compared to vehicle (*p* = 0.0181 and *p* = 0.0011, respectively, Fig. 4C), although not more than dexamethasone alone, in both responder and non-responder samples (Supplementary Fig. 3C). Cell viability after drug treatment for each patient is shown in Supplementary Fig. 3D.

Both dexamethasone and dasatinib induced cell cycle arrest in primary samples with more cells in G0 phase (*p* < 0.0001 and *p* = 0.0006 respectively), and less in G1-phase (*p* < 0.0001 and *p* = 0.0008) and S-phase (*p* = 0.0073 and *p* = 0.0046) (Supplementary Fig. 3E).

Thus, in primary BCP-ALL cells, GCR is highly expressed and maintains its developmental expression pattern, but expression level is not strictly correlated with sensitivity to glucocorticoids ex-vivo. While samples with lower levels of GCR in pro-B cells exhibited greater sensitivity to dexamethasone, a fraction of cells survived ex-vivo treatment in both responder and non-responder samples.

### Primary BCP-ALL cells that survive glucocorticoids are phenotypic pre-B cells

To deeply investigate the phenotype and signaling of dexamethasone-persistent cells, we classified these cells into their developmental state using our previously published approach^7,19^(see methods and gating strategy in Supplementary Fig. 4A). Compared to T-cells, which did not change after dexamethasone treatment (Supplementary Fig. 4B), dexamethasone-treated cells were significantly decreased in early pro-B (pro-BI, *p* = 0.0001) and early pre-B (pre-BI, *p* < 0.0001) populations but enriched in the late pre-B (pre-BII, *p* = 0.0047) (Fig. 4E). This late pre-B enrichment was observed in both dex-responders and non-responders (Supplementary Fig. 4C) although non-responders also had further enrichment in the Immature B population as well (*p* = 0.0151). Persistent cells demonstrated increased expression of CD45, a marker of hematopoietic maturity (Supplementary Fig. 4D, E) after dexamethasone treatment.

To understand pre-treatment cellular features that are associated with ex-vivo poor dexamethasone response we examined signaling differences in vehicle-treated cells between responder and non-responder samples focusing on the significantly different populations (pro-BII, pre-BI, and pre-BII cells). In the vehicle-treated condition, pro-BII and pre-BI cells from non-responders showed higher expression of targets normally transcriptionally repressed by the GCR, including GCR, cyclinB, cyclinA, pNFKB, and c-myc when compared to responders. Non-responders demonstrated higher pre-treatment levels of pc-ABL and lower levels of pCREB in pre-BII cells (Fig. 4F and Supplementary Fig. 5A). Following dexamethasone treatment, while responder samples significantly downregulated GCR pathway molecules (pNFKB, cyclinA, cyclinB) non-responders did not. These findings suggest that non-responders are insensitive to the repressive effects of GCs resulting in less cell death (Supplementary Fig. 5B, C). prpS6 and pCREB were upregulated in GC resistant pre-BII cells (*p* = 0.0466 and *p* = 0.0679 respectively, Fig. 4G). Similar trends were observed in pro-BII and pre-BI cells (Supplementary Fig. 5D, E). The addition of dasatinib to dexamethasone resulted in significant inhibition of several of these signaling targets (prpS6, pCREB, pSyk, and pSRC) in all populations (Fig. 4G and Supplementary Fig. 5D, E).

**Fig. 5:**
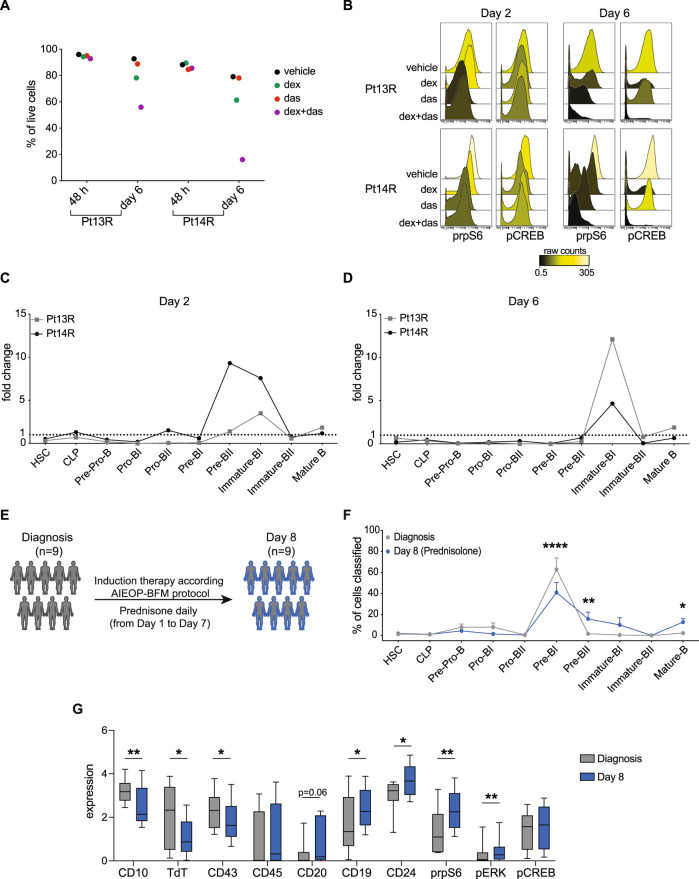
Prolonged treatment with glucocorticoids induces phenotypic plasticity in vitro and in vivo. **A** Percentage of live cells (cPARP^-^/cCASP3^-^) in vehicle (ethanol), dexamethasone (dex, 1 µM), dasatinib (das, 100 nM), and dexamethasone + dasatinib (dex + das) conditions in two primary samples following 48 h and 6 days of treatment. **B** Median prpS6 and pCREB expression in live cells in Pt13R (top) and Pt14R (bottom) at Day 2 and Day 6. **C** Fold change of events classified into B-cell developmental populations in dexamethasone condition compared to vehicle after 48 h of treatment. **D**. Fold change versus vehicle of cells classified into B-cell developmental populations in dexamethasone condition after 6 days of treatment. **E** Experimental workflow of cohort analyzed by CyTOF at diagnosis and day 8 following prednisone monotherapy as per AEIOP-BFM 2009 protocol (Supplementary Table 1). **F** Mean percentage of classified leukemic cells in nine matched samples at diagnosis (gray line) and day 8 (blue line) of treatment with prednisone. Asterisks indicate populations differentially abundant at day 8 compared to diagnosis based on ANOVA analysis followed by Fisher’s LSD test (α = 0.05). Pre-BI: *p* < 0.0001; Pre-BII: *p* = 0.0081; Mature-B: *p* = 0.0463. **G** Mean (with 5th and 95th percentile) protein expression of nine primary samples at diagnosis (gray bars) and day 8 (blue bars). Asterisks indicate significance based on a two-tailed paired *t*-test. CD10: *p* = 0.0024; TdT: *p* = 0.0176; CD43: *p* = 0.0385; CD19: *p* = 0.0436; CD24: *p* = 0.0313; prpS6: *p* = 0.0038; pERK: *p* = 0.0081. *p ≤ 0.5 **p ≤ 0.01; ***p ≤ 0.001. Source data are provided as a Source Data file.

Despite the impact of dasatinib on the active signaling characteristic of surviving cells, we did not observe increased apoptosis with the combination at this timepoint (Fig. 4C). To determine if prolonged signaling inhibition improves apoptosis in GC-resistant cells, two non-responder samples were treated with dasatinib and dexamethasone for 48 h and 6 days. In both samples, cells were resistant to any treatment after 48 h (Fig. 5A), accompanied by a minimal change in prpS6 or pCREB (Fig. 5B). Exposure at 6 days resulted in increased apoptosis in dexamethasone-treated cells compared to vehicle (14% in Pt13R and 18% in Pt14R) which was further increased when combined with dasatinib (37% in Pt13R and 63% in Pt14R) (Fig. 5A). At day 6, both prpS6 and pCREB were inhibited in response to the combination of dasatinib and dexamethasone (Fig. 5B).

Developmental classification of surviving cells at 48 h demonstrated enrichment of pre-BII (Pt14R) and immature-BI cells in both patients (Fig. 5C). By day 6, we observed further phenotypic maturity with both samples enriched (5-13 fold) in immature B-cells (CD19^+^/CD20^+^), supporting the hypothesis that dexamethasone-resistant cells differ from their initial phenotype (Fig. 5D). Given these results observed ex-vivo, next we wanted to understand if the same phenotypic and signaling shifts could be observed in vivo.

### Glucocorticoids modulate MRD cells towards mature B-cell states

To understand if the phenotype and signaling modulation induced by dexamethasone occur in patients receiving GCs, we analyzed minimal residual disease (MRD) from nine patients enrolled on the AIEOP/I-BFM clinical trial (NCT01117441) at diagnosis and day 8 by CyTOF^7,20^. Patients on this protocol received only prednisone during the first week of induction therapy; thus, these samples represent pre and post-GC timepoints (Fig. 5E).

Developmental classification of the leukemic cells demonstrated a reduction of pro-BI and pre-BI cells with a significant increase of pre-BII cells and mature B-cells at day 8 (Fig. 5F). Differential expression analysis performed on the pre-BII and immature populations revealed significantly increased expression of CD20, CD19, and CD24 (*p* = 0.063, *p* = 0.043, and *p* = 0.031) and decreased expression of CD10, CD43, and TdT (*p* = 0.002, *p* = 0.038, and *p* = 0.0117) at day 8. We observed activation of signaling in GC-treated patients with both prpS6 (*p* = 0.004) and pERK (*p* = 0.008) being highly expressed in the resistant cells (Fig. 5G). These in vivo findings are in line with our previous observations that GC-resistant BCP-ALL cells are more phenotypically mature compared to untreated cells and have activation of mTOR and PI3K signaling pathways.

### Targeting-activated signaling can overcome glucocorticoid resistance in vivo

**Fig. 6:**
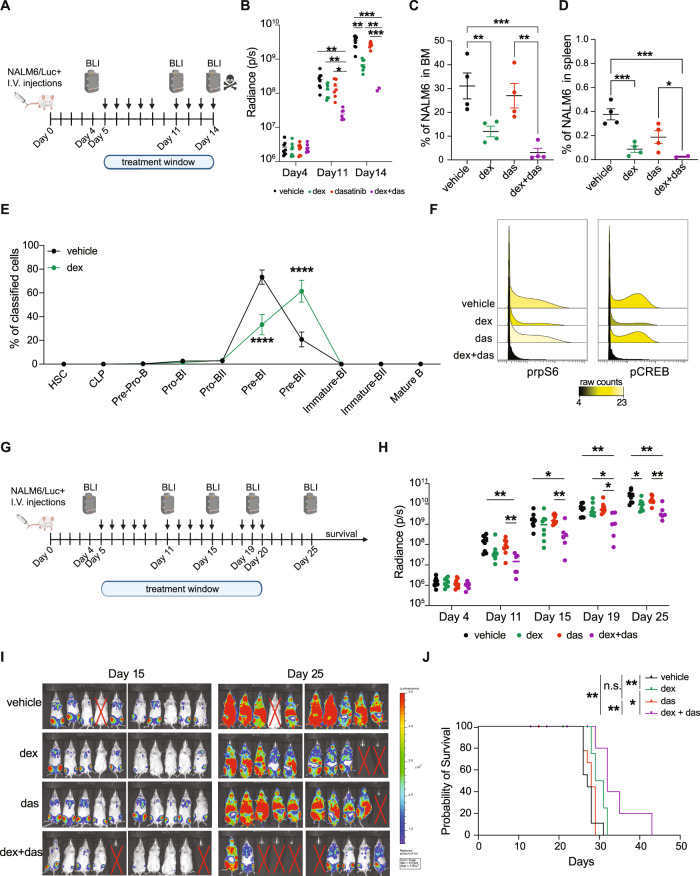
Combination of dexamethasone with dasatinib reduces engraftment and increases survival in vivo. **A** Experimental workflow **B** Tumor growth by bioluminescence. Dot plots represent the measured radiance for each mouse, while the bar indicates the mean radiance for each group. On day 4 *n* = 8 mice per group were measured, on day 11 *n* = 8 for vehicle and dexamethasone groups and *n* = 7 for dasatinib and dex + das groups, on day 14 *n* = 8 for vehicle and dexamethasone groups, *n* = 7 for dasatinib group and *n* = 2 for dex + das group. To test significance, a two-way ANOVA mixed model followed by Tukey’s test for multiple comparisons has been used with *p* = 0.05 confidence level. Day 11: dex+das vs veh *p* = 0.0077; dex vs dex + das *p* = 0.0087; das vs dex + das *p* = 0.0254. Day 14: dex vs veh *p* = 0.0028; dex + das vs veh *p* = 0.0009; dex vs dex + das *p* = 0.0012; das vs dex + das *p* < 0.0001. **C** Bone marrow engraftment of NALM6 cells (mean ± SEM), identified as mCD45.1^-^/ hCD19^+^ by flow cytometry (*n* = 4 mice per group) **D** Spleen engraftment of NALM6 cells (mean ± SEM), identified as mCD45.1^-^/ hCD19^+^ by flow cytometry (*n* = 4 per group except *n* = 2 in dexamethasone + dasatinib group). One-way ANOVA analysis followed by LSD’s test for statistics. **E** Developmental classification of engrafted cells (murine CD45.1^-^) in vehicle (*n* = 4) and dexamethasone-treated mice (*n* = 4) shown as mean ± SEM. Two-way ANOVA followed by Bonferroni correction was used to calculate significant comparisons. **F** pCREB and prpS6 in engrafted cells from mice treated with vehicle, dexamethasone, dasatinib, and dexamethasone + dasatinib. **G** Experimental workflow. **H** Tumor growth by bioluminescence. Dot plots and mean are represented, and a two-way ANOVA mixed model followed by Tukey’s test for multiple comparisons was used with *p* = 0.05 confidence level. Day 11: dex + das vs veh *p* = 0.0047; das vs dex + das *p* = 0.0069. Day 15: dex + das vs veh *p* = 0.0435; das vs dex + das *p* = 0.0060. Day 19: dex + das vs veh *p* = 0.0085; dex vs dex + das *p* = 0.0497; das vs dex + das *p* = 0.0142. Day 25: dex + das vs veh *p* = 0.0044; dex vs veh *p* = 0.0218; das vs dex + das *p* = 0.0019. **I** Bioluminescence images of NSG mice at Day 15 and Day 25 post engraftment with NALM6/Luc+ cells. Crossed-out mice indicate experimental mice excluded from the analysis because they did not develop leukemia or died without leukemia. **J** Kaplan–Meier analysis in vehicle, dexamethasone, dasatinib, or dexamethasone + dasatinib conditions. Significance by log-rank test for each group-group comparison. Dex vs veh: *p* = 0.0078; dex vs dex + das: *p* = 0.0376; das vs dex + das: *p* = 0.0029; dex + das vs veh: *p* = 0.0011. *p ≤ 0.5; **p ≤ 0.01; ***p ≤ 0.001. Source data are provided as a Source Data file. The experimental workflow was created with Biorender.com.

The addition of dasatinib to target active signaling in GC-resistant cells showed promising results in vitro. We next examined its utility in overcoming dexamethasone resistance in vivo (Fig. 6A). Combination treatment resulted in a significant reduction of engraftment compared to vehicle (*p* = 0.0077 Day 11 and *p* = 0.0009 Day 14), dexamethasone (*p* = 0.0087 Day 11 and *p* = 0.0012 Day 14) or dasatinib alone (*p* = 0.0254 Day 11 and *p* < 0.0001 Day 14) as assessed by BLI (Fig. 6B and Supplementary Fig. 6A). FACS analysis confirmed a decreased leukemic burden in a dexamethasone-treated group in bone marrow (*p* = 0.0003; Fig. 6C) and spleen (*p* = 0.0006; Fig. 6D) compared to the vehicle or dasatinib group (*p* = 0.0012 and *p* = 0.0483, respectively). Developmental classification of engrafted cells demonstrated that dexamethasone-resistant cells are enriched in the pre-BII population compared to vehicle-treated mice confirming our ex-vivo results and those in the MRD cohort (Fig. 6E). pCREB and prpS6 were reduced in cells from mice treated with the combination compared to vehicle or single drug (Fig. 6F).

A second experiment was performed with different treatment schedules to assess survival (Fig. 6G). Bioluminescence analysis confirmed that mice treated with the combination had significantly less leukemic burden at all timepoints (Fig. 6H, I and Supplementary Fig. 6B) compared to vehicle-treated mice. The combination group had significantly longer survival compared to vehicle (*p* = 0.0011), dexamethasone-only (*p* = 0.0376), and dasatinib-only (*p* = 0.0029) groups (Fig. 6J). Overall, this data suggests targeting the activated signaling can overcome GC resistance, in line with the ex-vivo data from primary samples and cell line models.

## Discussion

Glucocorticoids are a critical backbone of treatment for BCP-ALL^21^. Despite extensive clinical experience with GCs, the phenotypes of resistant cells and their mechanisms of resistance are not well understood. Studies examining the role of glucocorticoids and bone marrow B-lymphopoiesis have been primarily performed in mice where the expression of a functional GCR during healthy B-cell development has been shown^6^ as well as its relationship to B-cell transcription factors in the setting of leukemia^22^.

Here, analyzing normal human B-cell progenitors, we found a high correlation between the glucocorticoid pathway and B-cell development, specifically in the populations spanning the pro-B/pre-B checkpoint. In vitro treatment of healthy B-cell progenitors with GCs demonstrated cell cycle arrest in G0/G1 phases, suggesting a role for GCR signaling in cell cycle regulation across this checkpoint. Our prior work has highlighted the importance of this developmental stage and signaling in BCP-ALL relapse^7^. Further, GC-resistance in BCP-ALL is known to be predictive of treatment resistance and relapse^1,23^. Thus, these data suggest a link between a normal developmental role for GCR signaling and how it may be involved in GC resistance in BCP-ALL.

Understanding GC resistance and its relationship to treatment failure in BCP-ALL are of critical importance for patients. GC resistance has been linked to alterations in the gene encoding the GCR (*NR3C1)*^17,24,25^, as well as to mutations in other pathways, including apoptosis (*MCL-1*^26,27^, *BIM*^28^*, BCL-2*^29^)*, CELSR2*^16^, chromatin accessibility, *CREBBP*^29–31^, and glucose metabolism (*CNR2* and *TXNIP*^32^). More recently, multidrug resistance, including glucocorticoids, has been also linked to the ability of leukemia cells to attach to the stroma through integrins, and an inhibitor of α6 integrin has been tested to sensitize cells to chemotherapy^33^.

In general, sensitivity to GCs is thought to be dependent on GCR expression^34,35^. However, prior correlative studies from patients treated on BFM-based protocols showed no difference in GCR protein expression between good prednisone responders and poor prednisone responders when measured at diagnosis in bulk^36^. Here, analyzing GCR expression at the single-cell level, we found that patients with poor clinical prognoses and those we defined as poor GC responders ex-vivo expressed the highest levels of GCR in pro-B cells. Prior to treatment, these cells demonstrated activation of targets usually repressed by the GCR, including cyclinA, cyclinB, pNFKB, and c-myc, suggesting transcriptional deregulation in these cells.

Further, single-cell analysis of dexamethasone-treated cells revealed early pro-B cells being specifically targeted by the treatment, while late pre-B cells survived and were enriched. Recent studies in a different clinical condition, lymphopenia following stroke, also highlighted a preferential killing of B-cell progenitors with high doses of glucocorticoids^37^.

In leukemia, it has been previously described that GCs, highly used during induction therapy, induce a phenotypic modulation of leukemic cells with downregulation of progenitor markers such as CD34 and CD10 and upregulation of CD20. This has been investigated as a technical challenge in minimal residual disease detection^38,39^ for patients with BCP-ALL. Similar differentiation effects were observed in microarray studies of pediatric BCP-ALL samples treated with GCs^40^. In line with these data, we show the developmental nature of GCR signaling in early B-cell lymphopoiesis and that GC resistance in primary BCP-ALL cells is linked to the pre-B developmental stage. Kruth et al. previously described a double negative feedback loop between the PI3Kδ branch of the BCR pathway and GCR levels that can be interrupted through PI3Kδ inhibition^9^. Tonic pre-BCR signaling is required for the survival of pre-B cells and involves activation of proximal pre-BCR associated SRC family kinases such as Lyn, Fyn, Blk^41^ as well as spleen tyrosine kinase (SYK), which activates PI3K and MEK/ERK pathways^42,43^. Several kinase inhibitors of PI3K/AKT^44^, TCR^45^, MEK^46^, and JAK-STAT^47^ pathways have been used previously tested in combination with GCs in both B- and T-ALL, consistent with previous reports regarding the cross-talk between BCR pathway signaling and other pathways in B cells^48,49^.

In our primary cells and cell lines treated with dexamethasone, we found that GC-resistant cells, activate downstream survival pathways, including PI3K/mTOR and CREB signaling, and are responsive to the activation of the BCR. To inhibit this GC-resistant signaling, we tested four different tyrosine kinase inhibitors, including dasatinib (dual SRC/ABLi), ibrutinib (BTKi), trametinib (MEK1/MEK2i), and idelalisib (PI3Kδi). The combination of dasatinib with dexamethasone best inhibited the activated signaling in resistant cells. Dasatinib is used in combination with standard chemotherapy for patients with ABL alterations (Philadelphia positive^50^ or Ph-like BCP-ALL^51^). Several studies have recently investigated the possibility of using dasatinib to target pre-BCR^+^ leukemia with or without ABL alterations^52^, testing it either alone or in combination with small molecules disrupting CREB-binding protein (CBP)^53,54^. Here, we demonstrate its utility to target active signaling induced by steroid resistance resulting in increased apoptosis ex-vivo and improved survival in vivo.

Despite our findings, this study has several limitations to be addressed with future work. First, mechanistic studies in primary cells are limited due to the availability of materials and the heterogeneous nature of primary samples. To precisely dissect the cross-talk between the GCR pathway and relevant developmental signaling networks in early B cells, multi-omic approaches targeting protein and RNA simultaneously^55^, as well as genetic manipulation in cell lines of all candidate pathways is required.

Several studies of BCP-ALL, including our own, suggest the importance of its developmental origins in resistance to therapy^7,56,57^. Here, learning from the normal developmental stages of early human B-cells, we identified and characterized GC-resistant leukemic phenotypes and implicated pre-B-like leukemia cells with activated pre-BCR signaling to be GC-resistant. Targeting these cells by inhibition of this signaling with dasatinib may represent a therapeutic approach to overcome GC resistance.

## Methods

This research complies with all relevant ethical regulations. The use of the primary samples was approved by the Institutional Review Boards at the Pediatric Clinic of the University of Milano-Bicocca and at Lucile Packard Children’s Hospital at Stanford University. Animal studies were approved by Stanford Institutional Animal Care and Use Committee.

### Healthy donors and patient samples

Healthy human bone marrow samples (*n* = 3 for RNA-Seq, *n* = 4 for CyTOF) were purchased through AllCells. De-identified bone marrow samples from pediatric patients with BCP-ALL were obtained, under informed consent, from Lucile Packard Children’s Hospital at Stanford University (Stanford, CA, USA; *n* = 2) and from the Pediatric Clinic of University of Milano-Bicocca (Centro Maria Letizia Verga, Monza, Italy; *n* = 17). The use of these primary samples was approved by the Institutional Review Boards at both institutions. Written informed consent was obtained from the parents of the patients or their legal representatives who agreed to the use of biological material for research and clinical studies. To protect patients’ privacy, samples have been de-identified. Clinical data is provided in Supplementary Table 1.

### Cell lines

Human BCP-ALL cell lines, NALM6 and REH, were purchased from DMSZ (CRL-8286) and ATCC (#ACC 128), respectively, in December 2018 and November 2018. Frozen vials of early passages (within the third one) were used for the experiments. Cells were cultured in RPMI medium (Thermo Fisher Scientific) with 10% fetal bovine serum, 1% l-glutamine, and 1% penicillin/streptomycin at 37 °C in an incubator with 5% CO_2_. Cell lines have been regularly tested for *Mycoplasma* contamination and used for experiments within 5-10 passages from thawing.

### RNA extraction from sorted healthy populations

Mononuclear cells from three healthy donors were isolated by ficoll and depleted for myeloid and T-cell lineage populations as previously described in ref. ^10^. Briefly, cells were stained using biotin-conjugated antibodies listed in Supplementary Table 2 for 30 min, washed twice with CSM (PBS with 0.5% BSA and 0.02% sodium azide), and then incubated with BD Streptavidin Particles Plus (BD Biosciences) at manufacture’s recommended concentration for 30 min at room temperature. Particle-labeled cells were placed on a magnetic holder for 10 min, and the supernatant was collected and washed twice with CSM. Isolated cells were then stained for CD34(FITC)/CD24(BV421)/CD38(PE-Cy7) for 30 min at room temperature and washed once with CSM. Cells were then treated with 0.3% saponin for 15 min and stained intracellularly for TdT(APC) for 30 min. Stained cells were sorted on a FACS Aria in three populations (Fig. 1A): pre-pro-B (CD34^+^/CD38^+^/CD24^−^/TdT^+^), pro-B (CD34^low^/CD38^+^/CD24^+^/TdT^+^), pre-B (CD34^−^/CD38^+^/CD24^+^/TdT^-^) and collected in 1 mL of cell-staining media containing RNAase inhibitors (VRC, Promega; RNAsin, New England Biolabs) and RNA was extracted using RNeasy Micro kit (Qiagen) following manufacturer’s instructions.

### RNA sequencing analysis

RNA sequencing was performed on healthy bone marrow and BCP-ALL cell lines.

For healthy bone marrow, RNA samples were submitted for library preparation and sequencing to SFGF (Stanford Functional Genomics Facility). cDNA libraries were generated using the SMARTer Ultra Low kit (Takara Bio/Clonotech Laboratories) and a low-input library kit was used for library generation before being sequenced using Illumina HiSeq. Normalized counts were analyzed by using Ingenuity Pathway Analysis (Qiagen IPA) software. Comparison by trend was performed based on canonical pathways and upstream regulators to identify pathways upregulated from pre-pro-B to pro-B-cells and from pro-B to pre-B-cells using an insignificance threshold of 0.05. Linear regression was performed to identify pathways highly correlated with the most significant canonical pathway (BCR pathway) and upstream regulator (BCR complex) by using GraphPad Prism and *R*^2^ was calculated. All the pathways with *R*^2^ > 0.70 are listed in Supplementary Data 1.

Human BCP-ALL cell lines, NALM6, REHwt, and REH GCR, were treated with vehicle (ethanol) or dexamethasone (1 μM) for 48 h. Cells were collected for each condition, spun at 1500 rpm for 5 min, and washed twice with PBS. Afterward, RNA was extracted by using RNeasy Plus Micro Kit and RNA was sent to MedGenome Inc. (Foster City, CA, USA), where samples were tested for their purity using Agilent Bioanalyzer (RIN >8). All the samples passed QC standards before and after library preparation with the Illumina TruSeq mRNA stranded kit. Samples were sequenced on the NovaSeq instrument, 30 M paired reads (60 M total reads).

FASTQ files were first pre-processed using fastp to perform quality profiling, read filtering, and adapter trimming. Afterward, clean reads were aligned to the human reference genome (hg38) and salmon was used to quantify transcript abundance^58^. Then, the transcript level expression data were summarized to the gene level using tximport R package;^59^ low-expressed genes (less than 10) were removed before proceeding with differential expression analysis. DESeq2 package in R^60^ was used to calculate the differentially expressed genes between dexamethasone-treated cells and vehicle-treated ones. The log2FoldChange (log_2_FC), *p* values and adjusted *p* values (*p*adj) were calculated using default settings (alpha = 0.05, lfcThreshold = 1, pAdjustMethod = “BH”), and significant genes were considered the ones with *p*adj <0.05. All the differentially expressed genes in NALM6 and REH GCR, comparing dexamethasone-treated vs vehicle-treated cells, are listed in Supplementary Data 2. Volcano plots were generated using EnhancedVolcano R package^61^. Gene Set Enrichment Analysis (GSEA) was performed by GSEA software v4.1.0 (BROAD Institute—UC San Diego joint project) using default settings. The reactome database was used for the analysis and the normalized enrichment score (NES) was plotted by using GraphPad Prism.

### In vitro and ex-vivo cell perturbations

Healthy cells and primary leukemic cells were cultured in StemSpan SFEM serum-free media (Stemcell Technology) with vehicle (ethanol plus DMSO), dexamethasone (Sigma-Aldrich, #D9184) (1 µM), dasatinib (LC Laboratories, D-3307)(100 nM) or their combination for 48 h before being processed by CyTOF. For longer culture (6 days), primary cells were co-cultured with human bone marrow stroma in AIM-V serum-free medium (Gibco), as previously described in ref. ^62^. Briefly, human bone marrow immortalized stroma cells, obtained through a material transfer agreement with Prof. Dario Campana, were plated in 96-well plate at 2 × 10^4^ cells/well in RPMI media with 10% fetal bovine serum, 1% l-glutamine, 1% penicillin/streptomycin, and 10^−6^ M hydrocortisone (Sigma-Aldrich). After 4 days, when stromal cells reached confluence, each well was washed four times with PBS and four times with AIM-V medium before seeding 2 × 10^5^ leukemic cells per well resuspended in 200 μL of AIM-V media. After 6 days of co-culture, cells were retrieved by scraping each well with a 200-μL tip and were passed through a 1 mL syringe using a 19G needle. Cells were then used for downstream analysis using CyTOF.

Cell lines used for CyTOF and RNA-Seq experiments were treated in vitro using the same concentrations of primary cells. Trametinib (GSK1120212, #S2673), Ibrutinib (PCI-32765, #S2680), and Idelalisib (CAL-101, #S2226) were purchased by Selleckchem and used at the following final concentrations: Trametinib 100 nM, Ibrutinib 500 nM, and Idelalisib 500 nM for 48 h on REH GCR. For the pre-BCR crosslinking assay, REH GCR cells, previously treated for 48 h with vehicle, dexamethasone, or dasatinib, were stimulated with IgM at a final concentration of 900 μg/mL (Novus, NBP1-75017) for 10 min followed by H_2_O_2_ 0.5 M for 5 min.

### Retroviral generation of REH GCR and NALM6-Luc

The synthetic NR3C1 gene was expressed in a retroviral vector containing a GFP sequence cloned by Thermo Fisher Scientific (Waltham, MA, USA). Retroviral supernatant was produced, and transduction was performed in REH cells. About ~5 million 293GPs were added to 100 mm Poly-d-lysine-coated Petri dishes in complete DMEM media supplemented with 10% FBS, 10 mM HEPES, 2 mM GlutaMAX, 100 U/mL penicillin, and 100 μg/mL streptomycin (Gibco). The following day, cells were co-transfected with *NR3C1* vector plasmid and 4.5 μg RD114 with Lipofectamine 2000 (Invitrogen) in Opti-MEM media (Gibco). The media was replaced after 24 h and harvested at 48 and 72 h timepoints. The viral supernatant was frozen at −80 °C for long-term storage. For retroviral transductions, non-tissue culture treated six well plates were coated with 2 mL of 25 μg/mL retronectin (Takara) in PBS overnight, then blocked with 2% BSA in PBS for 25 min. About 1 mL of thawed retroviral supernatant was added to appropriate wells; plates were then centrifuged at 1800 RPM at 20 °C for 1 h and plates were placed in an incubator at 37 °C.

REH cells expressing GCR were enriched by the selection of GFP-positive cells using a BD FACSAria cell sorter. A similar protocol was used to generate NALM6 expressing luciferase. fLuc-tomato vector was cloned by Thermo Fisher Scientific (Waltham, MA, USA), and NALM6 cells were transduced with retroviral supernatant. NALM6 expressing luciferase were enriched by FACS sorting Tomato positive cells. Following sorting, cells were cultured in RPMI 10% FBS media as isogenic cells and tested for *Mycoplasma* contamination.

### Cell viability assay

NALM6 and REH GCR were treated with five different concentrations of dexamethasone (7.5, 15, 30, 60, and 120 nM for NALM6 cells and 1, 2.5, 5, 7.5, and 10 nM for REH GCR), five different concentrations of dasatinib (100, 150, 200, 250, and 300 nM for both cell lines), vehicle (ethanol + DMSO) and all their possible combinations (*n* = 36) for 48 h before being stained with Annexin V-APC Apoptosis Detection kit (Biolegend 640912, 420403, and 4220010) following manufacturer instructions and acquired on CytoFLEX Flow Cytometer (Beckman Coulter). A total of *n* = 4 biological replicates were performed, and the mean Bliss synergy score was calculated and plotted using SynergyFinder 3.0^63^.

### CyTOF analysis

Healthy bone marrow donors, leukemic primary cells, and cell lines were processed as previously described in refs. ^7,64^, stained with antibodies described in Supplementary Table 3 and analyzed using a Helios mass cytometer (Fluidigm). Specifically, the activation of pre-BCR was performed in NALM6 and REH GCR cells exposed to either vehicle (ethanol + DMSO) or treatments for 48 h. Cells were stimulated with anti-IgM antibody (Novus Biological #NBP1-75017, 9 μg/mL) for 10 min, followed by H_2_O_2_ (EMD Millipore #386790, 3.3 mM) for 5 min before being fixed with paraformaldehyde 1.6% for 10 min. For primary cells analysis, viably preserved bone marrow cells from children’s patients with BCP-ALL were thawed and resuspended in 90% RPMI medium (Thermo Fisher Scientific, Waltham, MA, USA) with 10% FBS, 0.025 U/mL Benzonase (Sigma-Aldrich), 1% l-glutamine, and 1% penicillin/streptomycin. Cells were rested at 37 °C for half an hour before in vitro perturbation for 48 h. Cells were fixed with paraformaldehyde 1.6% for 10 min at room temperature and barcoded using 20-plex palladium barcoding plates prepared in-house as described^65^. Briefly, cells were washed once with cell-staining medium (CSM; PBS with 0.5% BSA and 0.02% sodium azide), once with PBS, and once with PBS supplemented with 0.02% saponin and 0.02% sodium azide. Mass-tagged barcoding was then performed using a pattern of three of the six stable Palladium (Pd) isotopes (102, 104, 105, 106, 108, and 110) for each sample. Specifically, 5 μL of barcoding reagent was added into 1 mL of saponin buffer in a deep well block and resuspended. Following, 900 μL of the saponin containing the Pd isotopes was added to the cell suspension and incubated for 15 min at room temperature. To control for batch effects, one healthy bone marrow reference sample was included within each barcoding plate. Following barcoding, cells were washed with CSM, combined in a single tube, and stained for surface and intracellular markers as described in Supplementary Table 3. Before the surface staining, cells were treated with purified human Fc Receptor Binding Inhibitor (eBioscience) and then surface antibody mix was added to a final volume of 1 mL per barcoding plate. After 30 min of staining at room temperature, cells were washed with CSM, permeabilized with methanol 100% for 15 min at 4 °C, and stained with intracellular markers for 30 min in a final volume of 1 mL. Cells were then washed with CSM and stained with 1:5000 ^191^Ir/^193^Ir DNA intercalator (Fluidigm) in PBS with 1.6% PFA overnight at 4 °C. The following day cells were washed with CSM and ddH_2_O, filtered, and resuspended in ^139^La/^142^Pr/^59^Tb/^160^Tm/^175^Lu normalization beads before being analyzed using a Helios mass cytometer (Fluidigm) at 200 events/sec rate. IMD files were normalized with publicly available Matlab Normalizer v0.3^66^ (https://github.com/nolanlab/bead-normalization) and debarcoded using publicly available Matlab Single-cell Debarcoder v0.2^67^ (https://github.com/nolanlab/single-cell-debarcoder). Raw count values were transformed using the hyperbolic arcsine function with a cofactor of 5, before proceeding to analysis.

### Single-cell developmental classification

Primary leukemic cells were developmentally classified to the closest healthy B-cell population, as previously described in ref. ^7^. Briefly, live lineage negative (Lin^-^) cells (cPARP^-^/CD3^-^/CD33^-^) from healthy human bone marrow (hBM#7), included in each barcoding plate, were gated in ten developmental populations of normal B lymphopoiesis with Stanford Cytobank software (https://stanford.cytobank.org) using the gating strategy shown in Supplementary Fig. 4A. The distribution of each population was based on the expression of 9 B-cell developmental proteins that were used for the manual gating: CD45, CD20, CD34, CD38, CD179b, IgMi, TdT, and CD19.

Live Lin^-^ (cPARP^-^/CD3^-^/CD33^-^) B^+^ leukemic cells were then assigned to the most similar healthy fraction based on the shortest Mahalanobis distance among distances to all healthy developmental populations in these nine dimensions. A cell was designated as “unclassified” if none of the distances were below the classification threshold (Mahalanobis distance = 9). To control for batch effect, leukemic cells were always classified using the healthy BM control included in the same barcoding plate. The R script used for the B-cell developmental classification is publicly available (https://github.com/kara-davis-lab/DEVclassifier).

### In vivo experiments

NOD/SCID/IL2Rγ^−/−^ (NSG) mice were purchased from the Jackson Laboratory, housed, and treated under Stanford University Committee on Animal Welfare-approved protocol. Six-to-eight weeks old female mice were engrafted with 2.0 × 10^5^ NALM6-Luc^+^ cells via intravenous (I.V.) injection. When engraftment was detectable by bioluminescence imaging (BLI) (BLI > 0.5 × 10^6^), mice were randomized in four different experimental groups: vehicle, dexamethasone (American Reagent, Inc.), dasatinib (Eton Bioscience Inc # 100140013) and dexamethasone plus dasatinib. Two different in vivo experiments were performed. In the first one, *n* = 8 mice were used for each group to assess engraftment. Starting from day 5 until day 14 post engraftment, mice received 7.5 mg/kg (once/day) of dexamethasone via intraperitoneal (I.P.) injections, 35 mg/kg (twice/day) via oral gavage (O.G.) or vehicle (PBS I.P. and 60% PEG400, 40% water). At day 14, *n* = 4 mice per group were sacrificed, bone marrow and spleens were collected, stained with mCD45.1(FITC)/hCD19(BV421), and analyzed by FACS (Cytoflex, Beckman Coulter). Engraftment was assessed as the percentage of hCD19^+^/mCD45.1^−^ cells. Bioluminescence analysis was also performed at two different timepoints, day 11 and day 14. Mice treated with dexamethasone alone and in combination with dasatinib developed gastrointestinal toxicity, which resulted in dehydration and death unrelated to leukemia.

Thus, to assess survival, we modified the dosing regimen in the second experiment, in which *n* = 10 mice were used for each group. Starting from day 5 until day 20 post engraftment, mice received 5 mg/kg (once/day) of dexamethasone via I.P. injections, 25 mg/Kg (twice/day) via O.G. or vehicle (PBS I.P. and 50% PEG400, 50% water O.G.). Engraftment was monitored at day 11, day 15, day 19, and day 25 post NALM6 I.V. injections) by BLI and monitored for survival. Mice were sacrificed when clinical signs of leukemia were observed (hind limb paralysis), or they were moribund (mice reached BLI measurement of 10^11^ photons/sec). In the survival analysis, mice were censored if they were moribund for other reasons, such as GI toxicity (*n* = 2 in the dexamethasone group and *n* = 5 in a combined group), accidental death (*n* = 1 in the dasatinib group) or did not develop leukemia (*n* = 1 in vehicle group). Whensoever suspected leukemia-unrelated deaths occurred, FACS analysis of bone marrow and spleen were performed to confirm that death was not related to a high burden of leukemia (Supplementary Fig. 6C).

In vivo imaging was performed using a Spectrum IVIS instrument. Mice were anesthetized by isoflurane (2–3% in oxygen), were injected via I.P. with 100 mg/kg d-luciferin (Caliper Life Sciences, Hopkinton, MA), and 4 min later images were acquired with medium binning, beginning at 30 s exposure, or less if images were saturated. Images were analyzed with Living Image 4.7.3 software (PerkinElmer) and total flux measurement (photons/second) was quantified over the whole animal.

### Statistical analysis

Data analysis was performed using GraphPad Prism v9.3.1 software and R for RNA-Seq data. To test the statistical significance between the two groups, we applied a two-tailed unpaired or paired Student’s *t*-test. Comparisons between more than two groups was performed using one-way ANOVA when groups had equal variance or Krustal–Wallis test, nonparametric, when no equal variance could be assumed. For multiple comparison corrections we used Fisher LSD’s test to compare samples to a control group or Bonferroni/Tukey tests to compare all the experimental groups. Single-cell phosphoprotein expression was performed by using the Wilcoxon rank-sum test followed by the Bonferroni test for correction and −log of the adjusted *p* value has been plotted. Survival in animal experiments was represented by Kaplan–Meier curves, and significance was estimated by using a long-rank test. Significance is indicated according to conventional method: *p ≤ 0.5; **p ≤ 0.01; ***p ≤ 0.001; ****p ≤ 0.0001 and specified in each figure legend.

### Reporting summary

Further information on research design is available in the Nature Portfolio Reporting Summary linked to this article.

## Supplementary information

Supplementary Information

Reporting Summary

Description of Additional Supplementary Files

Supplementary Data 1

Supplementary Data 2

## Data Availability

Gene expression data generated for this study are publicly available in the GEO repository, accession number GSE214319. Mass cytometry data (annotated fcs.files) are available on FlowRepository, ID number FR-FCM-Z64E. Source data are provided as a Source Data file. The remaining data are available within the article, supplementary information, or source data file. Further correspondence and material requests should be addressed to Jolanda Sarno: jolanda@stanford.edu. Source data are provided with this paper.

## Source data

Source Data
