# Supplementary material for: Dasatinib overcomes glucocorticoid resistance in B-cell acute lymphoblastic leukemia
Source: Nat Commun. 2023 May 22;14:2935. doi: 10.1038/s41467-023-38456-y (PMC10203345; doi:10.1038/s41467-023-38456-y)
Supplement: Supplementary file 1 — Supplementary Information [file 41467_2023_38456_MOESM1_ESM.pdf]

Dasatinib Overcomes Glucocorticoid Resistance in B-cell Acute Lymphoblastic Leukemia

Jolanda Sarno[1,*], Pablo Domizi[1], Yuxuan Liu[1], Milton Merchant[1], Christina Bligaard Pedersen[2], Dorra Jedoui[1], Astraea Jager[1], Garry P. Nolan[4], Giuseppe Gaipa[3], Sean C. Bendall[4], Felice-Alessio Bava[5], Kara L. Davis[1,*]

[1] Hematology, Oncology, Stem Cell Transplant, and Regenerative Medicine, Department of Pediatrics, Stanford University, Stanford, CA, USA.
[2] Section for Bioinformatics, Department of Health Technology, Technical University of Denmark, Kongens Lyngby, Denmark;
[3] M. Tettamanti Research Center, Fondazione IRCSS San Gerardo dei Tintori, Monza (MB), Italy.
[4] Department of Pathology, Stanford University, Stanford, CA, USA.
[5] Baxter Laboratory, Department of Microbiology and Immunology, Stanford University, CA, USA.
[*] Correspondence: kardavis@stanford.edu (K.L.D); jolanda@stanford.edu (J.S.)

Supplementary Information

Supplementary Fig. 1

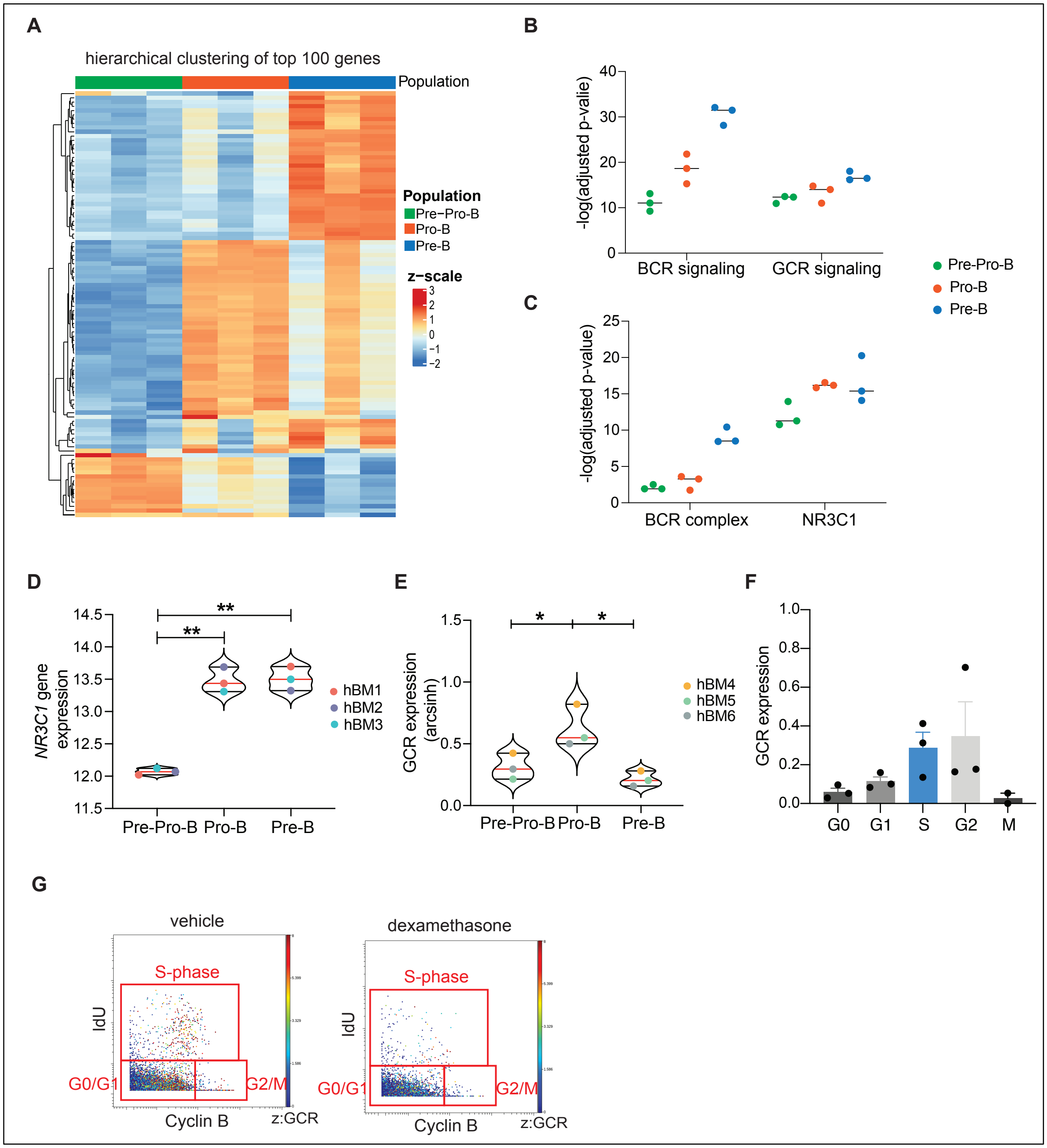

Supplementary Fig1. (A) Top 100 differentially expressed genes across Pre-Pro-B, Pro-B and Pre-B cells from three replicates colored according to the z-score in the legend. **(B)** IPA results of the -log (adjusted p-values) of each replicate (n=3 healthy donors) with the mean (bar) of BCR signaling compared to GCR signaling. Padj < 0.05 were considered significant and plotted **(C)** IPA results of the -log (adjusted p-values) of each replicate (n=3 healthy donors) with the mean (bar) of BCR complex compared to *NR3C1* gene. Padj < 0.05 were considered significant and plotted **(D)** *NR3C1* gene expression in pre-pro-B, pro-B and pre-B sorted cells, obtained from RNA-sequencing analysis. Asterisks indicate significance using two-tailed paired t-test. Pre-pro-B vs Pro-B p=0.0078; Pre-Pro-B vs Pre-B p=0.0074 **(E)** Glucocorticoid receptor (GCR) expression in pre-pro-B, pro-B and pre-B gated cells, obtained from CyTOF analysis. Asterisks indicate significance using two-tailed paired t-test. Pre-pro-B vs Pro-B p=0.0311; Pro-B vs Pre-B p=0.0243. Each dot color indicates the healthy donor. **(F)** Mean GCR expression (arcsinh transformed) after dexamethasone treatment in different cell cycle phases in n=3 healthy donors. **(G)** Dot plots show cell-cycle gating strategy (S, G0/G1 and G2/M) in vehicles and dexamethasone-treated cells. Plots are colored by GCR expression. Source data are provided as a Source Data file.

Supplementary Fig. 2

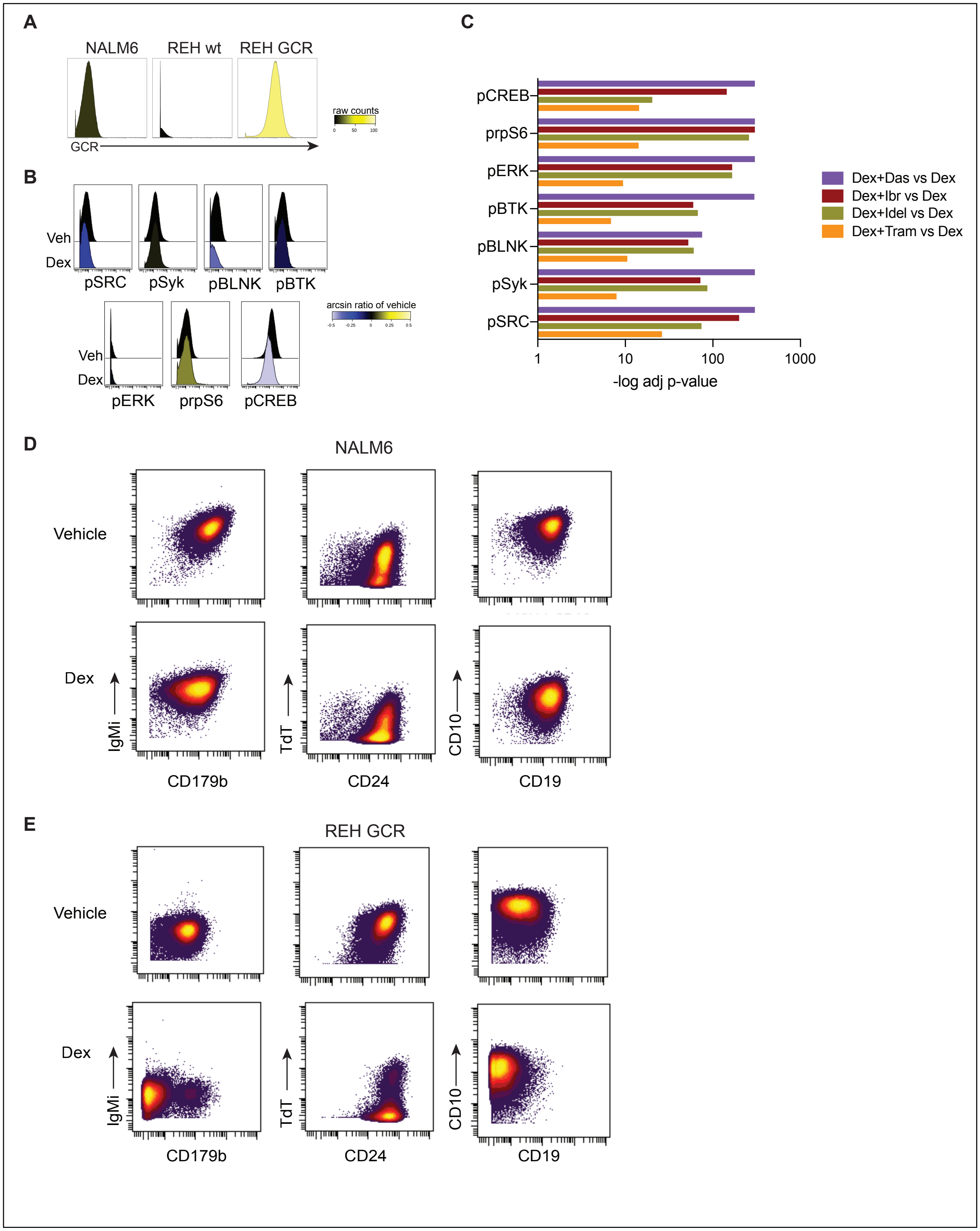

Supplementary Fig. 2 (A) Distribution of GCR expression in human BCP-ALL cell lines, NALM6, REH wt and REH GCR. **(B)** Marker expression in NALM6 cells treated with vehicle or dexamethasone for 48 hours. Histograms are colored based on mean expression compared to vehicle-treated cells. **(C)** Bar columns showing Wilcoxon rank test followed by Bonferroni correction for single cell analysis of the reported phosphoproteins in live cells following the treatments. The adjusted p-values refer to the comparison between combined treatments and dexamethasone alone treatment. Padj < 0.05 were considered significant and plotted **(D)** Phenotype of NALM6 cells in vehicle-treated and dexamethasone-treated cells. **(E)** Phenotype of REH GCR cells in vehicle-treated and dexamethasone-treated cells.
Dex: dexamethasone; das: dasatinib; Ibrut: Ibrutinib; Tram: Trametinib; Idel: Idelalisib
Source data are provided as a Source Data file.

Supplementary Fig. 3

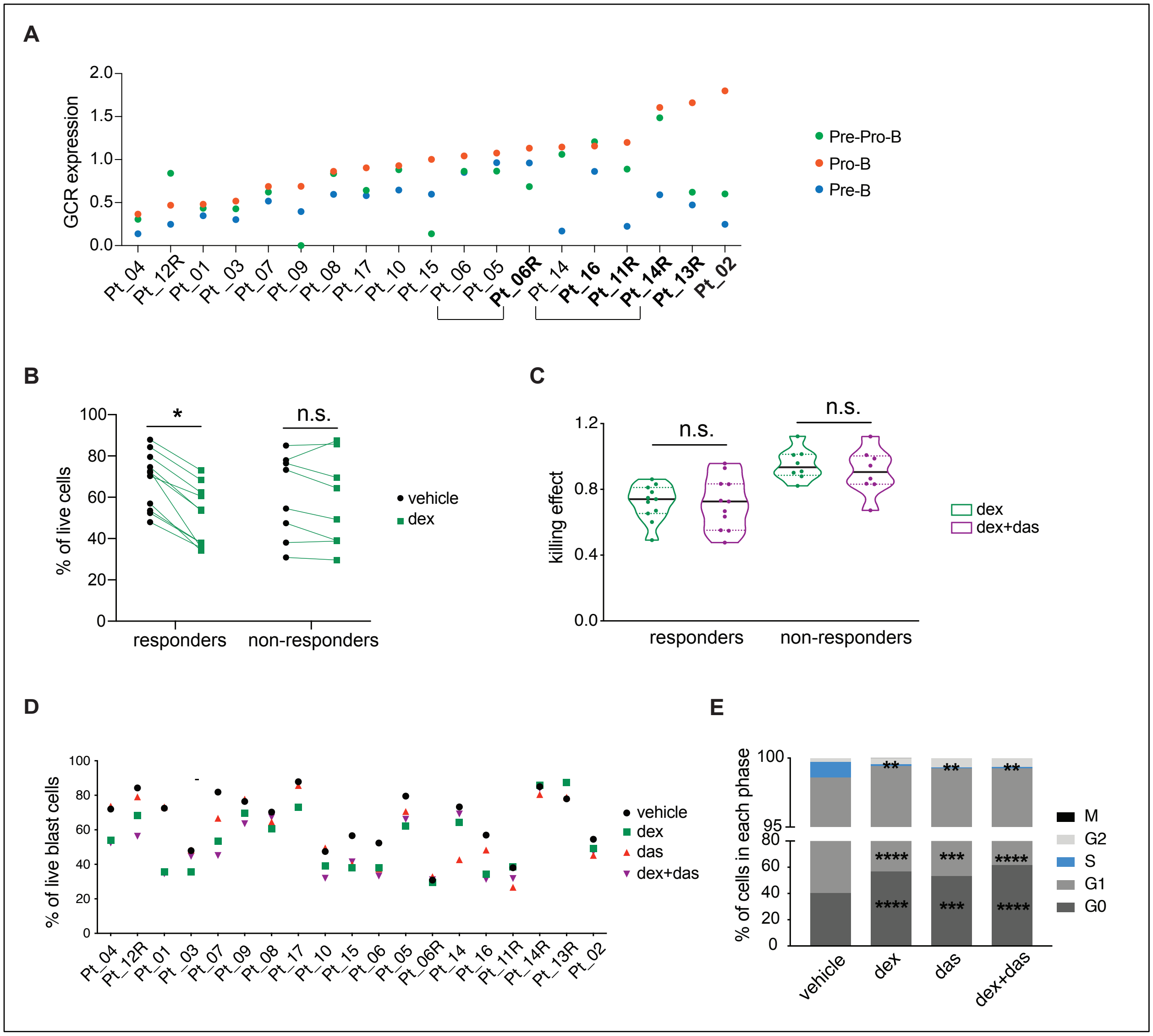

Supplementary Fig. 3 (A) GCR expression in gated pre-pro-B (CD34^{+}/CD38^{+}/TdT^{+}/CD24^{-}), pro-B (CD34^{low}/CD38^{+}/TdT^{+}/CD24^{+}) and pre-B (CD34^{-}/CD38^{+}/TdT^{-}/CD24^{+}) cells in the 19 primary samples analyzed by CyTOF. R indicates relapse samples and matched diagnosis/relapse samples are connected. In bold patients with poor prognostic features. Clinical information for the primary samples is summarized in Supplementary Table 1. **(B)** Percentage of live cells in dexamethasone responder (n=11 primary samples) and non-responder (n=8 primary samples) patients. Significance is calculated based on a two-tailed paired t-test. Responders: $p<0.0001$; Non-responders: $p=0.2924$. **(C)** Violin plots showing the killing effect of dex and dex+das treatments compared to vehicle-treated cells. Each dot represents a patient sample, dotted lines indicate 25% and 75% quartiles and bold lines indicate medians. Significance is calculated based on a two-tailed paired t-test. **(D)** Percentage of live cells of each patient sample after treatment with vehicle, dex, das and dex+das. **(E)** Mean percentage of each cell cycle phase in vehicle (ethanol), dex (1μM) das (100nM) and dex+das conditions in n=19 primary samples analyzed. Asterisks indicate p-values as calculated by a

two-tailed t-test, comparing each phase in the treated condition to the vehicle. Dex: G0 p<0.0001; G1 p<0.0001; S p=0.0074. Das: G0 p=0.0003; G1 p=0.0006; S p=0.0047; G2 p=0.0129; M p=0.0383. Dex+ das: G0 p<0.0001; G1 p<0.0001; S p=0.0058; G2 p=0.0155. dex: dexamethasone; das: dasatinib. * p ≤ 0.5 **p ≤ 0.01; *** p ≤ 0.001; **** p ≤ 0.0001; n.s = not significant. Source data are provided as a Source Data file.

Supplementary Fig. 4

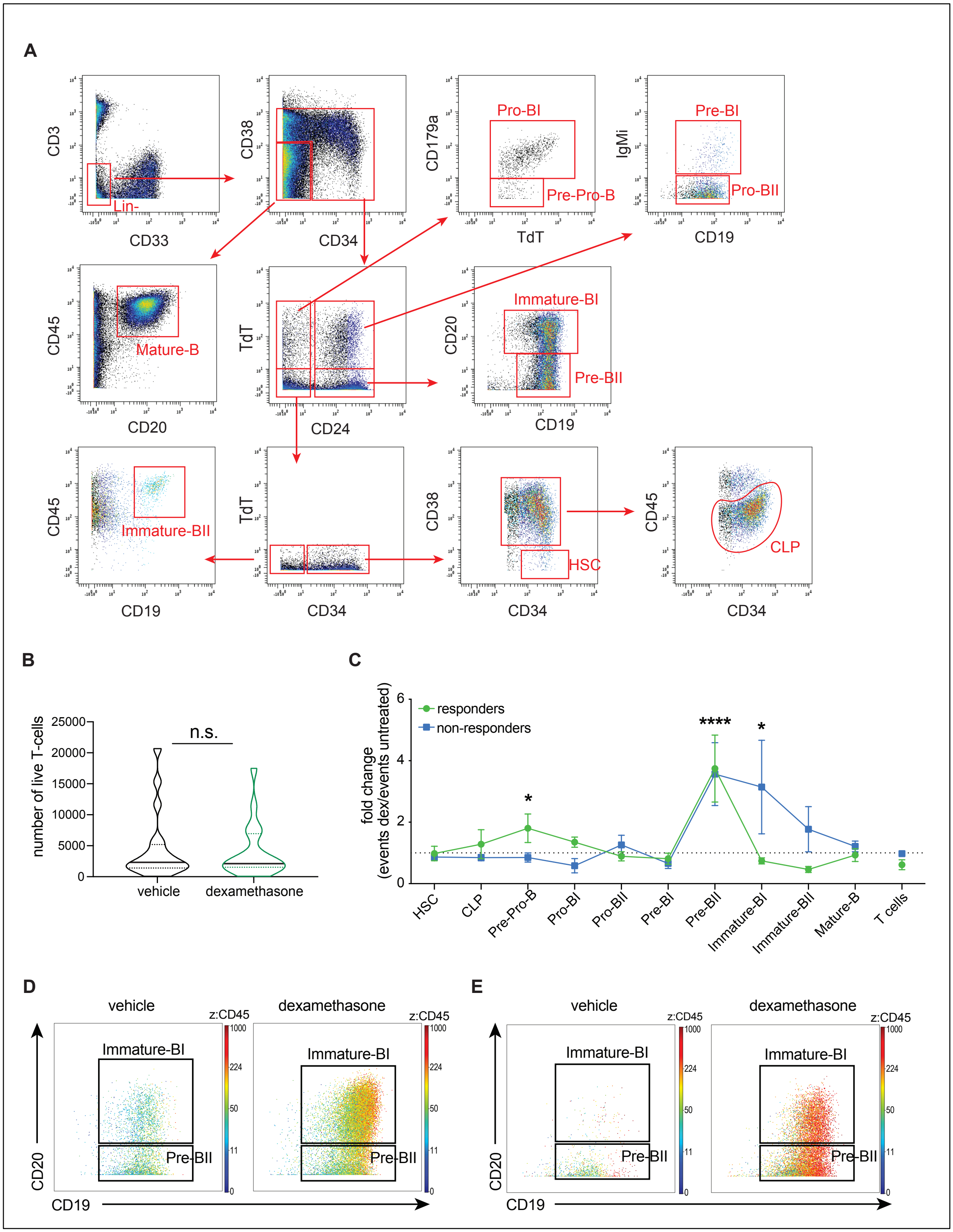

Supplementary Fig. 4 (A) Gating strategy used to gate healthy BM cells. The identified gated populations have been used as reference populations for B-cell developmental classification of leukemic cells. **(B)** Violin plots (dotted lines indicate 25% and 75% quartiles and bold lines indicate medians) of the absolute count of live T-cells (CD3+) in the n=19 primary samples analyzed after treatment with vehicle or dexamethasone. Two-tailed paired t-test was used to calculate statistics. **(C)** Mean fold change ± SEM of the events assigned to each population in dexamethasone-treated cells compared to vehicle, after B-cell developmental classification in responders (n=11 primary samples) and non-responders (n=8 primary samples). Responders and non-responders groups are shown and ANOVA analysis followed by LSD's test (α=0.05) has been performed to identify populations statistically significant from the T-cell population. Responders: Pre-Pro-B p=0.0432; Pre-BII p<0.0001. Non-responders: Pre-BII p=0.0039; Immature-BI p=0.0151. **(D)** Pre-BII and Immature BI cells in the vehicle and dexamethasone-treated conditions in a representative responder patient. **(E)** Pre-BII and Immature BI cells in vehicle and dexamethasone-treated conditions in a representative non-responder patient. Plots are colored based on the expression of CD45.
* p ≤ 0.5 **p ≤ 0.01; *** p ≤ 0.001; **** p ≤ 0.0001; n.s = not significant Source data are provided as a Source Data file.

Supplementary Fig. 5

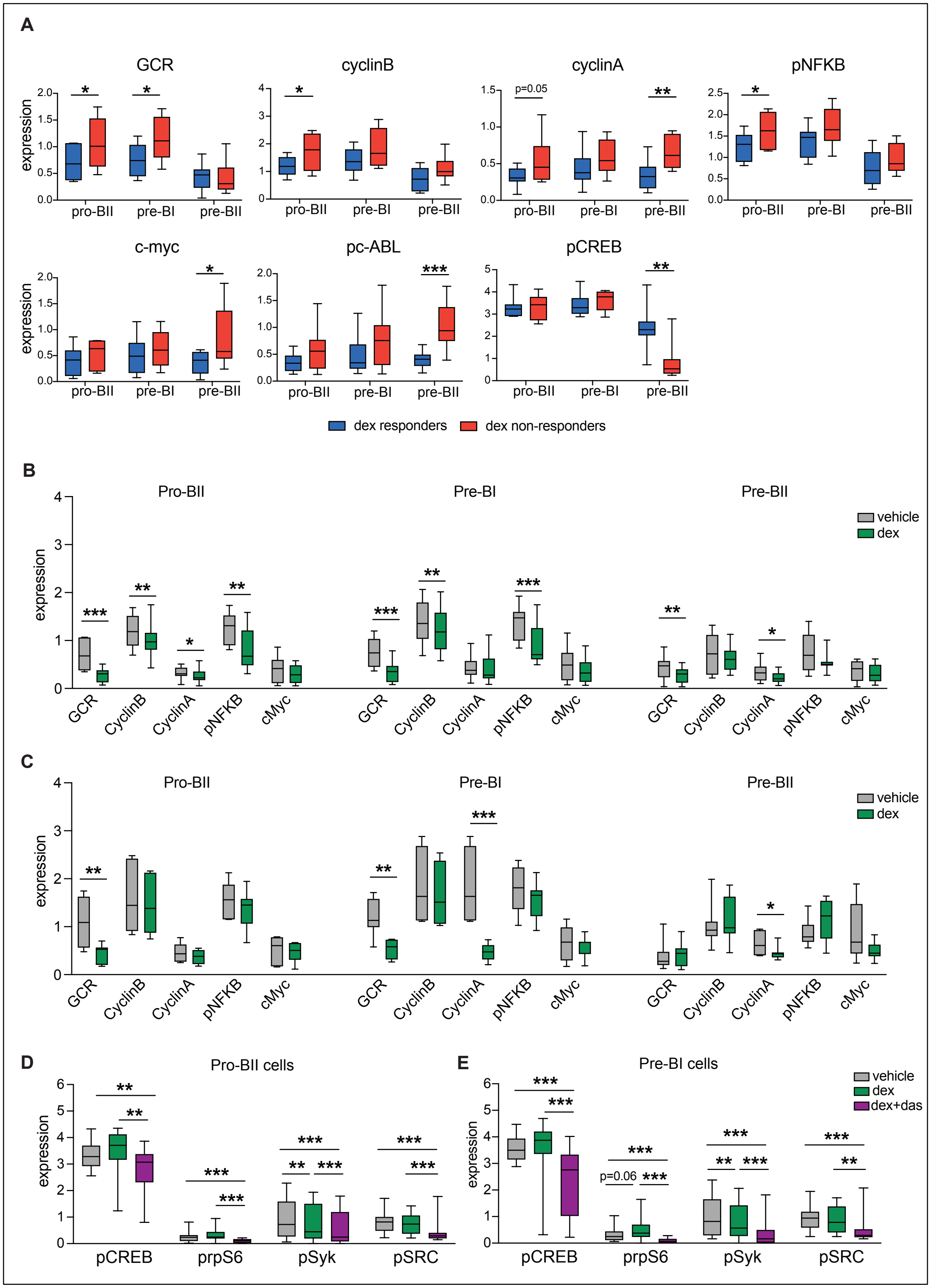

Supplementary Fig. 5 (A) Signaling protein expression levels in dex responders (n=11) and dex non-responders (n=8) patients. Box bars indicate mean expression, error bars the 5th and 95th percentile and asterisks indicate significant differences based on a two-tailed unpaired t-test. GCR: Pro-BII p=0.0316; Pre-BI p= 0.0214. CyclinB: Pro-BII p=0.0368. CyclinA: Pre-BII p=0.0024. pNFKB: Pro-BII p=0.0312. c-myc: Pre-BII p=0.0231. pc-Abl: Pre-BII p=0.0005. pCREB: Pre-BII p=0.0010 **(B)** Protein expression levels in vehicle and dexamethasone (dex) treated cells in responder patients (n=11 primary samples). Box bars indicate mean expression, error bars the 5th and 95th percentile and asterisks indicate significant differences based on a two-tailed paired t-test. Pro-BII: GCR p<0.0001; CyclinB p=0.0090; CyclinA p=0.0134; pNFKB p=0.0010. Pre-BI: GCR p<0.0001; CyclinB p=0.0053; pNFKB p<0.0001. Pre-BII: GCR p=0.0045; CyclinA p=0.0446. **(C)** Protein expression levels in vehicle and dexamethasone (dex) treated cells in non-responder patients (n=8 primary samples). Each protein is measured in pro-BII, pre-BI and pre-BII classified populations. Box bars indicate mean expression, error bars the 5th and 95th percentile and asterisks indicate significant differences based on a two-tailed paired t-test. Pro-BII: GCR p=0.0089; CyclinB p=0.0942. Pre-BI: GCR p=0.0040; CyclinA p=0.0008. Pre-BII: CyclinA p=0.0224. **(D)** Protein expression levels in the vehicle, dexamethasone and combined conditions in pro-BII classified cells. Box bars indicate mean expression in the 18 primary samples, error bars the 5th and 95th percentile and asterisks indicate significant differences based on a two-tailed paired t-test followed by Bonferroni correction (α=0.05). pCREB: dex vs dex+das p=0.0021; dex+das vs veh p=0.0013. prpS6: dex vs dex+das p=0.0001; dex+das vs veh p=0.0006. pSyk: dex vs veh p=0.0066; dex vs dex+das p=0.0008; dex+das vs veh p<0.0001. pSRC: dex vs dex+das p=0.0007; dex+das vs veh p<0.0001. Pt10 was excluded from the analysis because no cells were classified in this population following treatment. **(E)** Protein expression levels in the vehicle, dexamethasone and combined conditions in pre-BI classified cells. Box bars indicate mean expression in the 18 primary samples, error bars the 5th and 95th percentile and asterisks indicate significant differences based on a two-tailed paired t-test followed by Bonferroni correction (α=0.05). pCREB: dex vs dex+das p=0.0003; dex+das vs veh p=0.0004. prpS6: dex vs dex+das p=0.0003; dex+das vs veh p=0.0006. pSyk: dex vs veh p=0.0085; dex vs dex+das p<0.0001; dex+das vs veh p<0.0001. pSRC: dex vs dex+das p=0.0010; dex+das vs veh p<0.0001. Pt10 was excluded from the analysis because no cells were classified in this population following treatment.
* p ≤ 0.5 ** p ≤ 0.01; *** p ≤ 0.001. Source data are provided as a Source Data file.

Supplementary Fig. 6

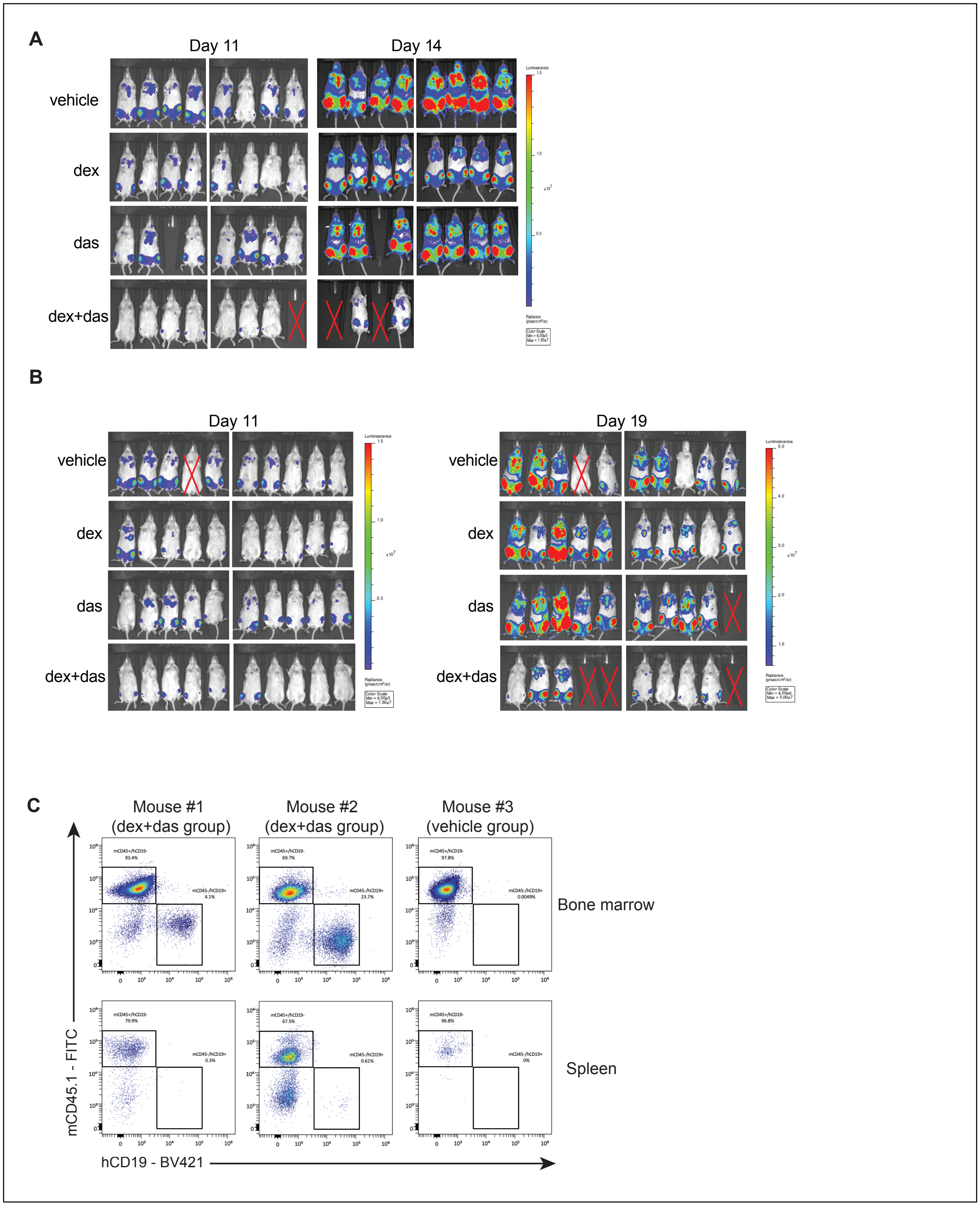

Supplementary Figure 6. (A) Bioluminescence images of NSG mice at Day 11 and Day 14 post engraftment with NALM6/Luc+ cells. Images were acquired with IVIS Spectrum instruments and are colored based on a radiance scale from 6.00 e^5 to 1.50 e^7 p/sec/cm^2/sr. **(B)** Bioluminescence images of NSG mice at Day 11 and Day 19 post engraftment with NALM6/Luc+ cells.**(C)** Representative plots of engraftment assessment in mice censored from the survival experiment due to leukemia unrelated death. Mouse#1 and #2 belonged to dex+das group and they died due to opportunistic infection on day 13 and day 17 post-engraftment. Mouse #3 belonged to the vehicle group and was censored since did not develop leukemia. Plots represent the absence of leukemia at the endpoint of the experiment, day 43.

Supplementary Table 1. Clinical information, cytogenetics and prednisone response data of BCP-ALL patients.

Patient ID	Prognostic Translocation	Treatment Protocol	Prednisone Response	Sample analyzed
Pt 1	t(12;21)	AIEOP2000	PGR	diagnosis
Pt 2	t(9;22)	AIEOP2000	PPR	diagnosis
Pt 3	negative	AIEOP2000	PGR	diagnosis
Pt 4	t(12;21)	AIEOP2000	PGR	diagnosis
Pt 5	negative	AIEOP2000	PGR	diagnosis
Pt 6	negative	AIEOP2000	PGR	diagnosis and relapse
Pt 7	negative	AIEOP2000	PGR	diagnosis
Pt 8	negative	AIEOP2000	PGR	diagnosis
Pt 9	negative	AIEOP2000	PGR	diagnosis
Pt 10	t(1;19)	AIEOP2000	PGR	diagnosis
Pt 11	negative	AIEOP2000	PGR	relapse
Pt 12	t(12;21)	AIEOP2000	PGR	relapse
Pt 13	negative	AIEOP2000	PGR	relapse
Pt 14	negative	AIEOP2000	PGR	diagnosis and relapse
Pt 15	negative	AIEOP2009	PPR	diagnosis
Pt 16	*MLLr*	COG AALL0631	n.a.	diagnosis
Pt 17	*MLLr*	COG AALL0631	n.a.	diagnosis
Pt 18	t(12;21)	AIEOP2009	PGR	diagnosis and day 8
Pt 19	t(9;22)	AIEOP2009	PPR	diagnosis and day 8
Pt 20	negative	AIEOP2009	PGR	diagnosis and day 8
Pt 21	t(12;21)	AIEOP2009	PGR	diagnosis and day 8
Pt 22	t(12;21)	AIEOP2009	PGR	diagnosis and day 8
Pt 23	negative	AIEOP2009	PGR	diagnosis and day 8
Pt 24	*CRLF2r*	AIEOP2009	PGR	diagnosis and day 8
Pt 25	*CRLF2r*	AIEOP2009	PGR	diagnosis and day 8
Pt 26	*CRLF2r*	AIEOP2009	PGR	diagnosis and day 8

Patients samples from 1 to 15 were obtained from Pediatric Clinic University of Milano-Bicocca (Monza, Italy). Patients samples 16 and 17 were obtained from Lucile Packard Children's Hospital at Stanford (Stanford, CA, USA). Patients data from 18 to 26 were taken from previously published cohorts[1,2].
Sex of the analyzed patients: 11 males, 10 females, and 5 n.a.
Median age at diagnosis: 5.8 years old (range 1 to 17).
PGR: prednisone good responder, PPR: prednisone poor responder as assessed in NCT00430118 and NCT01117441 clinical trials. n.a. not available.

Supplementary Table 2. List of antibodies used for lineage depletion of healthy bone marrow donors used for RNA-Seq experiment.

Antibody	Clone	Manufacturer	Final Concentration
CD16	3G8	Biolegend	2 ug/mL
CD14	HCD-14	Biolegend	2 ug/mL
CD11c	3.9	Biolegend	2 ug/mL
CD56	HCD56	Biolegend	2 ug/mL
CD3	UCHT1	Biolegend	2 ug/mL

Supplementary Table 3. CyTOF panel used for healthy bone marrow, cell lines and primary samples analyses.

Protein	Epitope	Clone	Manufacturer	Lot number	Metal Isotope	Concentration (µg/mL)	Staining
CD45		HI30	Biolegend	304002	Y89	2	S
IdU					I127		
cPARP	cleaved	F21-852	BD	552597	La139	2	I
pSRC	pY418	K98-37	BD	custom	Pr141	2	I
CD19		HIB19	Biolegend	302202	Nd142	0.5	S
CD7		CD7-6B7	Biolegend	343102	Nd143	0.5	S
CD22*		HIB22	Biolegend	302502	Nd143	1	S
IKAROS	total	D10E5	CST	9034BF	Nd145	2	I
CD8a		RPA-T8	Fluidigm	3146001B	Nd146	2	S
CD20		2H7	Biolegend	302302	Sm147	2	S
CD34		581	Fluidigm	3148001B	Nd148	1	S
pNFkβ	pS529	K10-895.12.50	BD	558393	Sm149	1	I
CEBPα*	total	16C12B70	Biolegend	662102	Sm149	1	I
hsp90	total	AC88	Abcam	ab13492	Nd150	0.5	I
GCR	total	D8H2	CST	3660BF	Eu151	4	I
pAkt	pS473	D9E	CST	4060BF	Sm152	4	I
IgMi	total	polyclonal	Novus	NBP1-75017	Eu153	0.25	I
cyclinA	total	BF683	BD	554175	Sm154	2	I
kappa*		MHK-49	Biolegend	316502	Sm154	1	S
lambda*		MHL-38	Biolegend	316602	Sm154	1	S
pSTAT5	pY694	47	BD	custom	Gd155	1	I
CD10		HI10a	Biolegend	312202	Gd156	1	S
PU.1	total	9G7	CST	2258BF	Gd157	4	I
CD179b	total	HSL11	Biolegend	349802	Gd158	1	I
Bcl2	total	100	Biolegend	658702	Tb159	2	I
CD24		ML5	Biolegend	311102	Gd160	2	S
c-myc	total	D84C12	CST	5605BF	Dy161	1	I
Casp3	cleaved	C92-605	BD	custom		2	I
CD127		A019D5	Fluidigm	3149011B	Dy162	1	S
TdT	total	E17-1519	BD	custom	Dy163	2	I
cyclinB	total	GNS-1	BD	554177	Dy164	2	I
PAX5	total	1H9	Biolegend	649702	Ho165	0.5	I
pRb	pS807/811	J112-906	BD	558389	Er166	2	I
CD43		CD43-10G7	Biolegend	343202	Er167	2	S
CD38		HIT2	Biolegend	303502	Er168	1	S
CD33		WM53	BioLegend	303419	Tm169	2	S
CD3		UCHT1	Biolegend	300414	Er170	2	S
BAX	total	6A7	Novus	28566		2	I
pSyk	pY319/pY353	17a	BD	custom	Yb171	4	I
prpS6	pS235/236	N7-548	BD	custom	Yb172	1	I
pErk	pT202/pY204	D13-14-4E	CST	4370BF	Yb173	2	I
pCREB	pS133	87G3	CST	9198BF	Yb174	3	I
pc-abl	pY393	19H22L15	ThermoFisher	702334	Yb175	2	I
pHH3	pS28	HTA28	Biolegend	641002	Yb176	2	I

S: surface staining; I: intracellular staining. * Indicates antibodies used in cell lines experiments as replacement of respective antibodies conjugated with the same metal isotope.

References

1. Good, Z. et al. Single-cell developmental classification of B cell precursor acute lymphoblastic leukemia at diagnosis reveals predictors of relapse. Nat. Med. 24, 474–483 (2018).
2. Sarno, J. et al. SRC/ABL inhibition disrupts CRLF2-driven signaling to induce cell death in B-cell acute lymphoblastic leukemia. Oncotarget 9, (2018).
