# Supplementary material for: Dasatinib overcomes glucocorticoid resistance in B-cell acute lymphoblastic leukemia
Source: Nat Commun. 2023 May 22;14:2935. doi: 10.1038/s41467-023-38456-y (PMC10203345; doi:10.1038/s41467-023-38456-y)
Supplement: Supplementary file 2 — Reporting Summary [file 41467_2023_38456_MOESM2_ESM.pdf]

nature portfolio

Corresponding author(s): Kara Davis, Jolanda Sarno

Last updated by author(s): Mar 14, 2023

Reporting Summary

Nature Portfolio wishes to improve the reproducibility of the work that we publish. This form provides structure for consistency and transparency in reporting. For further information on Nature Portfolio policies, see our Editorial Policies and the Editorial Policy Checklist.

Statistics

For all statistical analyses, confirm that the following items are present in the figure legend, table legend, main text, or Methods section.

n/a	Confirmed	
☐	☒	The exact sample size (n) for each experimental group/condition, given as a discrete number and unit of measurement
☐	☒	A statement on whether measurements were taken from distinct samples or whether the same sample was measured repeatedly
☐	☒	The statistical test(s) used AND whether they are one- or two-sided<br>*Only common tests should be described solely by name; describe more complex techniques in the Methods section.*
☒	☐	A description of all covariates tested
☐	☒	A description of any assumptions or corrections, such as tests of normality and adjustment for multiple comparisons
☐	☒	A full description of the statistical parameters including central tendency (e.g. means) or other basic estimates (e.g. regression coefficient) AND variation (e.g. standard deviation) or associated estimates of uncertainty (e.g. confidence intervals)
☐	☒	For null hypothesis testing, the test statistic (e.g. F, t, r) with confidence intervals, effect sizes, degrees of freedom and P value noted<br>*Give P values as exact values whenever suitable.*
☒	☐	For Bayesian analysis, information on the choice of priors and Markov chain Monte Carlo settings
☒	☐	For hierarchical and complex designs, identification of the appropriate level for tests and full reporting of outcomes
☒	☐	Estimates of effect sizes (e.g. Cohen's d, Pearson's r), indicating how they were calculated

Our web collection on statistics for biologists contains articles on many of the points above.

Software and code

Policy information about availability of computer code

Data collection	Flow Cytometry and Sorting: BD Facs DIVA, Cytexpert.<br>Mass Cytometry: Helios Instrument Control Software v6-7.
Data analysis	Mass Cytometry: Cytobank (Stanford server) from 8.0 to 9.4 versions. R package for developmental classifier (https://github.com/kara-davis-lab/DEVclassifier)<br>RNA-Seq expression analysis: GSEA 4.1.0, GEO, IPA (October 2020 release)<br>Prism GraphPad (versions 7 to 9).<br>Microsoft Excel 16.63.1<br>Synergy scores: SynergyFinder 3.0<br>In vivo imaging: Living Image 4.7.3.

For manuscripts utilizing custom algorithms or software that are central to the research but not yet described in published literature, software must be made available to editors and reviewers. We strongly encourage code deposition in a community repository (e.g. GitHub). See the Nature Portfolio guidelines for submitting code & software for further information.

Data

Policy information about availability of data

All manuscripts must include a data availability statement. This statement should provide the following information, where applicable:

- Accession codes, unique identifiers, or web links for publicly available datasets
- A description of any restrictions on data availability
- For clinical datasets or third party data, please ensure that the statement adheres to our policy

A data availability statement has been added to the main text. Specifically, gene expression data are publicly available in the GEO repository, accession number GSE214319. Mass cytometry data (annotated fcs.files) are available on FlowRepository, ID number FR-FCM-Z64E.

Human research participants

Policy information about studies involving human research participants and Sex and Gender in Research.

Reporting on sex and gender	This information has been reported in Supplementary Table 1, when available. All the analyses performed were not gender biased.
Population characteristics	In Supplementary Table 1, for each patient genetic background and prednisone response according clinical trial definition and age at diagnosis have been reported, when available.
Recruitment	Samples were obtained from patients enrolled in AIEOP2000, AIEOP2009 and COG ALL 0631 clinical trials. The samples were collected as part of IRB-approved clinical research studies.
Ethics oversight	The Institutional Review Board (IRB) of Stanford University and University of Milano-Bicocca approved the use of these primary samples for this work.

Note that full information on the approval of the study protocol must also be provided in the manuscript.

Field-specific reporting

Please select the one below that is the best fit for your research. If you are not sure, read the appropriate sections before making your selection.

☒ Life sciences ☐ Behavioural & social sciences ☐ Ecological, evolutionary & environmental sciences

For a reference copy of the document with all sections, see nature.com/documents/nr-reporting-summary-flat.pdf

Life sciences study design

All studies must disclose on these points even when the disclosure is negative.

Sample size	Sample size of primary samples was determined based on availability of samples with sufficient material and annotated clinical data. For mice experiment, assuming medium effect (f=0.5) in the drug treated groups 10 mice per group were used to reach a 80% power using a one-site test at a=0.05.
Data exclusions	No data were excluded from the analysis.
Replication	Replicates of the primary samples by mass cytometry were not performed sistematically. Only 2 primary samples were replicated to generate data in Figure 4C and 5A. Replicates for the healthy RNA-Seq analysis were obtained from 3 different healthy individuals aged matched. All in vitro cell lines experiments were performed in biological replicates (three or four).
Randomization	For in vivo experiments, mice were randomized in four different experimental groups based on bioluminiscence data acquired at day 4 post-injection.
Blinding	Primary patient samples were run in different plates and the label assignment was required to investigate batch effect. Therefore, blinding was not possible for such analysis. Flow cytometry and drug treatments were performed by the same investigator so blinding was not possible. Mice imaging instead was performed by investigator blinded to the group allocation.

Reporting for specific materials, systems and methods

We require information from authors about some types of materials, experimental systems and methods used in many studies. Here, indicate whether each material, system or method listed is relevant to your study. If you are not sure if a list item applies to your research, read the appropriate section before selecting a response.

Materials & experimental systems

n/a	Involved in the study	
☐	☒	Antibodies
☐	☒	Eukaryotic cell lines
☒	☐	Palaeontology and archaeology
☐	☒	Animals and other organisms
☐	☒	Clinical data
☒	☐	Dual use research of concern

Methods

n/a	Involved in the study	
☒	☐	ChIP-seq
☐	☒	Flow cytometry
☒	☐	MRI-based neuroimaging

Antibodies

Antibodies used

All the antibodies used for mass cytometry experiments with informations regarding epitope, clone, vendor, lot number, metal isotope conjugated and concentrations used are described in Supplementary Table 3.
For flow cytometry antibodies:
Antigen, clone, manufacturer, fluorophore, concentration used, cat number
- CD34, 581, BD Bioscience, FITC, 5 uL, 555281
- CD24, ML5, BD Bioscience, BV421, 5 uL, 562789
- CD38, HB7, BD Bioscience, PE-Cy7, 5 uL, 335790
- TdT, E17-1519, BD Bioscience, APC, 10 uL, 347210.
- Annexin V, n/a, Biolegend, APC, 1 uL, 640920.
- 7AAD, n/a, Biolegend, 1 uL, 420403.
- mCD45.1, A20, Biolegend, FITC, 2uL, 110706
- hCD19, HIB19, Biolegend, BV421, 2uL, 302234.

Validation

All antibodies were validated in human cells (cell lines or primary cells) known to be positive or negative controls for a given antibody target. Each antibody was titrated in concentration range from 0.5 to 8 ug/mL and the lowest concentration that discriminate positive from negative without splill over in another channels was chosen.

Eukaryotic cell lines

Policy information about cell lines and Sex and Gender in Research

Cell line source(s)

DMSZ and ATCC

Authentication

Cells lines were purchased in November (REH) and December (NALM6) 2018. Frozen vials of early passages (within the third one) were thawed and used within 5-10 passages for experiments.

Mycoplasma contamination

Cell lines were routinely tested for Mycoplasma contamination before in vitro experiments using MycoAlert Mycoplasma Detection kit from Lonza and were confirmed to be negative.

Commonly misidentified lines
(See ICLAC register)

Name any commonly misidentified cell lines used in the study and provide a rationale for their use.

Animals and other research organisms

Policy information about studies involving animals; ARRIVE guidelines recommended for reporting animal research, and Sex and Gender in Research

Laboratory animals

NOD.Cg-Prkdcscid Il2rgtm1Wjl/SzJ (NSG, #:005557) mice 6 to 8 weeks old were purchased from the Jackson Laboratory. Mice were house in pathogen-free condition with temperature range 19 C to 21 C, humidity range 45 - 65%, 11.5 light and 12.5 dark hours.

Wild animals

The study did not involve wild animals.

Reporting on sex

Female mice were used for all the experiments.

Field-collected samples

The study did not involve field-collected samples.

Ethics oversight

All the animal experiments were approved by Animal Care Committee of Stanford University. The APLAC protocol #32878 was revised yearly and the number of animals used approved by ethics committee.

Note that full information on the approval of the study protocol must also be provided in the manuscript.

Clinical data

Policy information about clinical studies
All manuscripts should comply with the ICMJEguidelines for publication of clinical research and a completedCONSORT checklist must be included with all submissions.

Clinical trial registration	AIEOP ALL 2000 (ClinicalTrials.gov Identifier: NCT00613457)<br>AIEOP ALL 2009 (ClinicalTrials.gov Identifier: NCT01117441)<br>COG ALL 0631
Study protocol	the full protocol can be accessible on ClinicalTrials.gov
Data collection	Primary samples with available material were collected at diagnosis or relapse of the disease and used for this study
Outcomes	We did not report any outcome status for this work

Flow Cytometry

Plots

Confirm that:

- [x] The axis labels state the marker and fluorochrome used (e.g. CD4-FITC).
- [x] The axis scales are clearly visible. Include numbers along axes only for bottom left plot of group (a 'group' is an analysis of identical markers).
- [x] All plots are contour plots with outliers or pseudocolor plots.
- [x] A numerical value for number of cells or percentage (with statistics) is provided.

Methodology

Sample preparation	For healthy donors sorting: Following lineage depletion, using antibodies listed in Supplementary Table 2 as previously described, cells were stained for CD34(FITC)/CD24(BV421)/CD38(PE-Cy7), treated with 0.3% saponin for 15 minutes and then stained intracellular for TdT(APC). Cells were sorted on a FACS Aria in 3 populations: pre-pro-B (CD34+/CD38+/CD24-/TdT+), pro-B (CD34low/CD38+/CD24+/TdT+), pre-B (CD34-/CD38+/CD24+/TdT-) and collected in 1mL of cell staining media containing RNAase inhibitors.<br>For cell lines cell viability assay: cells were stained with Annexin V-APC Apoptosis Detection kit following manufacturer instructions.<br>For mice experiments, bone marrow cells were isolated and filtered using 100 uM mesh cell strainer while spleen cells were filtered using 70uM mesh cell strainer. Samples were then depleted of red blood cells using ACK buffer for 5' and then washed in staining media (PBS 2% FBS) twice before being stained with mCD45.1 and hCD19 for 15 minutes at room temperature in dark. After the staining cells were washed with staining media and analyzed.
Instrument	For sorting, cells were sorted on FACS Aria II (BD Bioscience), while as FACS analyzer we used Cytoflex (Beckman Coulter). Single stains and FMOs of each fluorophore were used for cytometry setup and gating.
Software	Analyses were performed using FACS Diva, Cytobank Premium (Stanford server) and OMIQ
Cell population abundance	Sorted populations were re-run to check purity following sorting and purity was consistently greater than 90%.
Gating strategy	Unstained and FMOs were used for setting gating strategy and define gates.<br>For the sorting, using FSC and SSC we defined lymphocytes. In the CD34/CD38 biaxal plot CD34+ with or without CD38 expression were chosen to then define sorting populations on CD24 and TdT biaxal plot. 3 populations were sorted pre-pro-B (CD34+/CD38+/CD24-/TdT+), pro-B (CD34low/CD38+/CD24+/TdT+), pre-B (CD34-/CD38+/CD24+/TdT-).<br>For cell viability assay unstained control as well as untreated controls were used to define the gate of live and dead gates and the Annexin V by 7AAD plot was used to define live cells (Ann V -/ 7AAD -).<br>For the in vivo experiments, lymphocytes from human and murine cells were gated using FSC and SSC and then human cells (mCD45.1-/hCD19+) were distinguished from murine cells (mCD45.1+/hCD19-).

- [x] Tick this box to confirm that a figure exemplifying the gating strategy is provided in the Supplementary Information.
