# Supplementary material for: Dasatinib overcomes glucocorticoid resistance in B-cell acute lymphoblastic leukemia
Source: Nat Commun. 2023 May 22;14:2935. doi: 10.1038/s41467-023-38456-y (PMC10203345; doi:10.1038/s41467-023-38456-y)
Supplement: Supplementary file 3 — Description of Additional Supplementary Files [file 41467_2023_38456_MOESM3_ESM.pdf]

Description of Additional Supplementary Files

Supplementary Data 1
Description: Ingenuity Pathway Analysis (IPA) p-values indicating the statistical significance of the enrichment of genes in each pathway obtained from RNASeq of Pre-Pro-B, Pro-B, Pre-B sorted population in each replicate. Comparison by trend was performed to identify the most significant upregulated pathways from Pre-ProB to Pro-B and Pre-B. R2 column indicates the linear regression score of pathways correlated with BCR signaling pathway and BCR complex. All the pathways with R2 > 0.70 are listed. Bold font indicates pathways plotted in Figure 1B-C.

Supplementary Data 2
Description: Differentially expressed genes obtained from RNA-Seq comparing using DESeq2 package NALM6 and REH GCR cells treated with dexamethasone (1μM) for 48 hours compared to vehicle-treated (ethanol) cells. The log2FoldChange (log2FC), p-values and adjusted p-values (padj) were calculated using default settings (alpha = .05, lfcThreshold = 1, pAdjustMethod = "BH"), and significant genes were considered the ones with padj < 0.05.
